# Remote sensing of savanna woody species diversity: A systematic review of data types and assessment methods

**DOI:** 10.1371/journal.pone.0278529

**Published:** 2022-12-01

**Authors:** Emmanuel Fundisi, Solomon G. Tesfamichael, Fethi Ahmed

**Affiliations:** 1 Department of Geography, Environmental Management and Energy Studies, University of Johannesburg, Johannesburg, South Africa; 2 Geospatial Analytics, eResearch Knowledge Centre, Human Sciences Research Council, Pretoria, South Africa; 3 School of Geography, Archaeology and Environmental Studies, University of Witwatersrand, Johannesburg, South Africa; Universidade Federal de Uberlandia, BRAZIL

## Abstract

Despite savannas being known for their relatively sparse vegetation coverage compared to other vegetation ecosystems, they harbour functionally diverse vegetation forms. Savannas are affected by climate variability and anthropogenic factors, resulting in changes in woody plant species compositions. Monitoring woody plant species diversity is therefore important to inform sustainable biodiversity management. Remote sensing techniques are used as an alternative approach to labour-intensive field-based inventories, to assess savanna biodiversity. The aim of this paper is to review studies that applied remote sensing to assess woody plant species diversity in savanna environments. The paper first provides a brief account of the spatial distribution of savanna environments around the globe. Thereafter, it briefly defines categorical classification and continuous-scale species diversity assessment approaches for savanna woody plant estimation. The core review section divides previous remote sensing studies into categorical classification and continuous-scale assessment approaches. Within each division, optical, Radio Detection And Ranging (RADAR) and Light Detection and Ranging (LiDAR) remote sensing as applied to savanna woody species diversity is reviewed. This is followed by a discussion on multi-sensor applications to estimate woody plant species diversity in savanna. We recommend that future research efforts should focus strongly on routine application of optical, RADAR and LiDAR remote sensing of physiologically similar woody plant species in savannas, as well as on extending these methodological approaches to other vegetation environments.

## 1. Introduction

Savanna biomes are characterised by marked wet and dry seasons, with monthly mean temperatures ranging between 20 and 30°C [1] and annual rainfall ranging between 200 and 1350 mm [2]. These climatic conditions along with other factors such as fire and herbivory promote heterogeneous environments that are composed of varying extents of grassland, herbaceous cover and woody plant species [3–5]. Around the globe, there are different types of savannas, comprising of (i) Tropical−Subtropical, (ii) Temperate, (iii) Mediterranean, (iv) Flooded and (v) Montane savanna [6]. Tropical−Subtropical savannas are mostly distributed near the equator, and bordered by tropical rainforests and deserts [3]. Temperate savannas are found in mid-latitude regions with semi-arid to semi-humid climate, and dominated by grass and shrubs [7]. Mediterranean savannas are also found in mid-latitude and Mediterranean environments, and are dominated by shrubs and small evergreen trees [8]. Flooded savannas are located in tropics and Subtropical regions, and consist of large expanses of flooded grasslands either seasonally or throughout the year [9]. Montane savannas are classified according to geographical regions they are located in, for instance Tropical, Subtropical, Temperate and are typically situated in high altitude areas [10].

Savanna woody plants serve as a source of primary productivity, by providing food for humans, livestock and wildlife [11–13]. Savanna ecosystems also play a pivotal role in regulating global climate dynamics [14–16]. However, climate change coupled with anthropogenic activities continue to contribute towards high spatial and temporal variations in woody plant species diversity, composition and productivity in the savanna environment [17, 18]. Studying the phenology of savanna vegetation, [19] reported exacerbated spatial and temporal variations of savanna vegetation due to modified weather patterns as a result of climate change.

Climate projections indicate that hotter, drier conditions will continue to intensify across the savanna ecosystems [3, 12, 20–22]. Such climatic changes are expected to increase the dominance of certain plant species in the ecosystem [18, 23]. It is therefore important to have timely information about plant species diversity and associated dynamics of woody plant species in order to design and implement sustainable management strategies for savanna ecosystems [24]. However, assessment of woody plant species diversity has largely relied on traditional methods, which are generally costly and time-consuming [11, 25–27].

Remote sensing offers efficient assessment methods at considerably lower costs than traditional field-based surveys [28–30]. Several studies have applied remote sensing systems to assess woody plant species diversity in different savanna environments and reported solid performances [31–36]. Given the proliferation of works applying remote sensing to savanna environments, a number of authors have attempted to compile reviews of such works [13, 24, 37–42].

For example, [13] conducted a review of remote sensing applications to savanna environments at a global scale. They reported that low plant cover and limited background reflection of herbaceous plants and grassland impacted classification of woody plant species. Since Brazil is home to large expanses of savanna vegetation (Cerrado) in South America, multiple studies have reviewed the application of remote sensing to that savanna environment [40, 43, 44].

[40] conducted a systematic and integrative review of studies on vegetation composition in Brazilian Passive Restoration and Active Restoration sites. The authors found deficiencies, including studies being focused on single areas, resulting in insufficient studies across boundaries of tropical regions that comprise different forest types. [39] reviewed perspectives of applying remotely sensed and field-based data in the mapping of fragmented forests in the tropical savanna. Their study noted the need for rapid and cost-effective assessments of tree species diversity and forest structure.

Research has shown that increases in climate variability in Africa, which has approximately 65% savanna coverage by area has had an impact on the savanna ecosystem in terms of photosynthetic activity, abscission and the length of growing-season [21, 23, 45]. [37] conducted a systematic review of vegetation phenology in Africa, and classified studies based on the methods and techniques used. Their review stressed the need for finer spatial resolution satellite sensors for regional phenological assessments. Such a review study showed progress in remote sensing of savanna environments and underscored the need to review studies beyond those concerned with phenological assessments in savanna regions. [24] reviewed the application of remote sensing in analysing vegetation in the Sudano-Sahelian savanna zone between 1975 and 2014. They noted that remote sensing applications largely emphasise mapping broad vegetation types or distinct vegetation forms.

Considering the rapidly improving classification methods and data qualities, it is important to track the status of species diversity assessments using remote sensing. The present study, therefore, reviews the literature by placing focus on two aspects of savanna woody species diversity assessment using remote sensing. Firstly, it aims to look into diversity assessment techniques by grouping them into categorical classification and continuous-scale assessment methods. Secondly, it seeks to review applications through the lens of remote sensing data types including optical (multispectral and hyperspectral) and structural (Radio Detection And Ranging (RADAR) and Light Detection and Ranging (LiDAR)) systems.

## 2. Literature survey method and structure of the review

**Fig 1:**
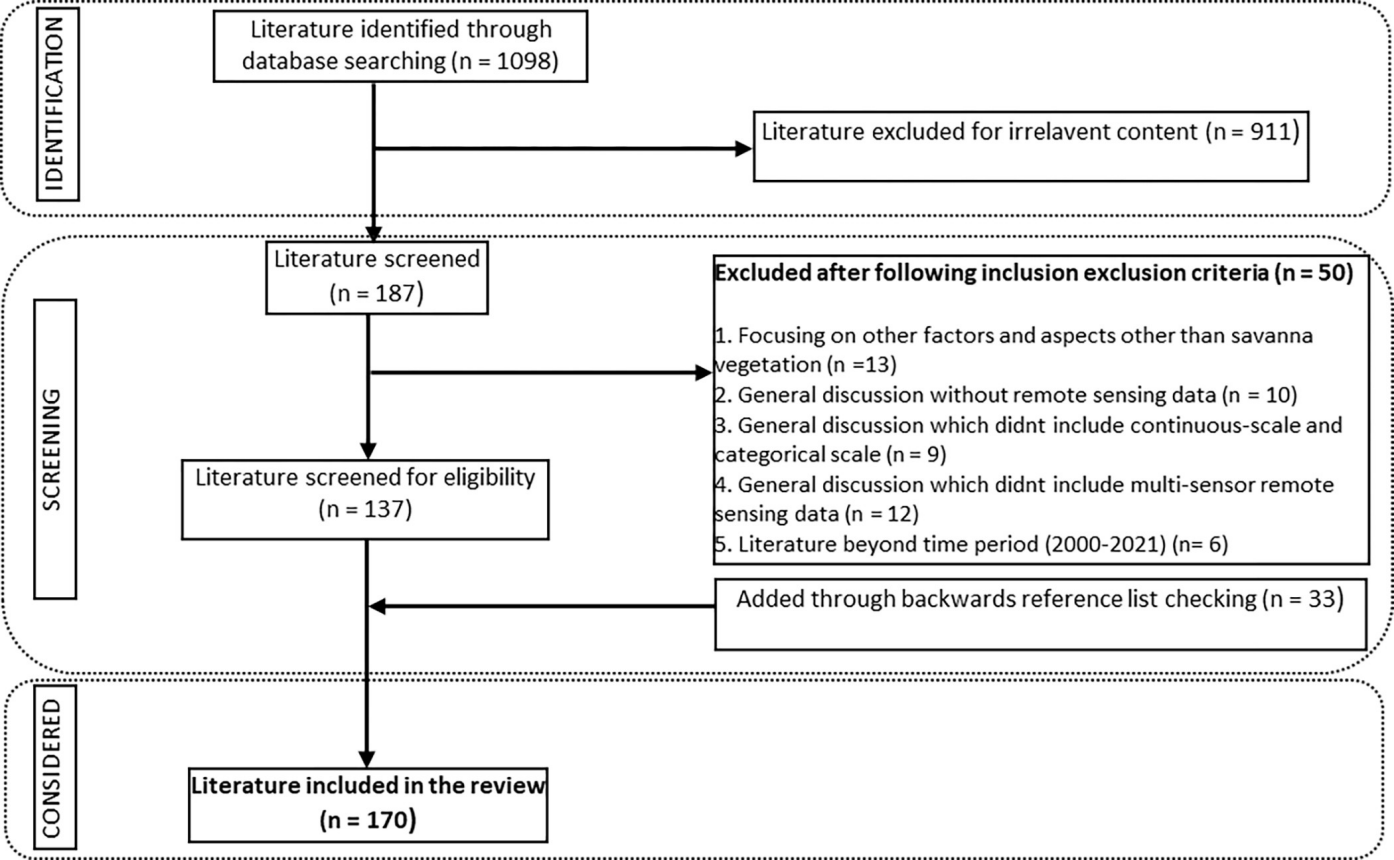
Literature selection process using Preferred Reporting Items for Systematic Reviews and Meta-Analyses (PRISMA) framework.

Literature was searched from seven bibliographic databases, including Web of Science, Science Direct, Scopus, Taylor and Francis Online, SpringerLink, IEEE Xplore and Academic Search Ultimate. The search used catch phrases such as “remote sensing of savanna woody plant species diversity”, “classification of savanna woody plant species”, “remote sensing application for species discrimination”, “statistical analysis of savanna woody plant species using remote sensing”, “multi-sensor remote sensing for savanna woody plant species diversity”, “optical—RADAR, optical—LiDAR and LiDAR—RADAR data fusion for savanna plant species diversity estimation”. Adopting the Preferred Reporting Items for Systematic Reviews and Meta-Analyses (PRISMA) framework (Fig 1), [46] the study identified 1098 documents (including books and scientific research articles) that were potentially relevant to this review study. Of these, 911 were omitted initially due to limited relevance to the review. The omission resulted in 187, which were further screened following the inclusion-exclusion criterion (Fig 1) and resulted in 137 articles that had strong significance to the review. During analysis of the information in the 137 articles and the writing process, we re-incorporated 33 articles resulting in a total of 170 (articles) used for core review of the paper. Please notice that this number does not include sources that were used to introduce generic concepts outside of remote sensing.

The review is organised as follows. Section 3 provides a brief introduction of general ecological discrimination of species using categorical-scale and continuous-scale approaches. Section 4 focuses on remote sensing applications to savanna woody plant species diversity using classification/categorical scale approaches. Section 5 reviews studies that applied remote sensing to savanna woody plant species diversity using continuous-scale assessment techniques. Section 6 is dedicated to reviewing studies that applied multi-source remote sensing for savanna woody plant species discrimination using categorical-scale and continuous-scale approaches. Section 7 concludes the review by addressing the benefits, challenges and future potential of remote sensing based monitoring of woody plant species diversity in savanna environments.

## 3. Species discrimination using categorical-scale and continuous-scale approaches

Categorical-scale discrimination of species adopts the principle of allocating different species into distinct classes [47]. In that respect, categorical-scale measurement seeks to establish a correspondence between observed classes with mutually exclusive attributes. Numerous research in the field of forestry and ecology have exhausted this approach more specifically in enumerating species diversity. Though this approach is the most favoured information extraction method, it can under-or-overestimate the number of species that potentially exist on the ground [48]. In contrast, continuous-scale assessment/modelling converts unique species data into a continuous diversity scale, thus allowing stretching the ability to estimate as many species as possible. Continuous-scale assessment approach eliminates the restriction on the number of species that can be identified in a given area [49, 50]. This is achieved by applying statistical indices such as the Shannon diversity index [50], Species richness [51] and Simpson’s Index [52]. Continuous-scale measurement has largely been applied in ecology and biodiversity assessment, for quantifying species distribution in an area. It is therefore an indicator of diversity level without placing emphasis on the type of categories.

## 4. Remote sensing of savanna woody plant species diversity using categorical-scale approach

### 4.1 Categorical classification of savanna woody plant species using optical remote sensing

Optical images typically use sensors that operate in the visible (0.4–0.7 μm) and infrared (0.7–1.3 μm) regions of the electromagnetic spectrum to acquire images of the Earth’s surface [53, 54]. These regions are sensitive to biochemical variations that exist in plant foliage [13]. While multispectral data (e.g., Landsat, Sentinel) are widely available and used for species classification, they are inefficient in discriminating subtle differences between plant characteristics. In contrast, hyperspectral data that use several narrow and contiguous bands provide improved classification capability even at the species level [54]. One of the significant advantages of optical data (particularly multispectral data) is that, it has a long history of data acquisition allowing for time series analysis that is vital for monitoring the temporal dynamics of biodiversity [24, 55, 56]. Common weaknesses of optical remotely sensed data include applications being limited to ideal weather conditions particularly for land cover and species discrimination purposes. Opportune weather conditions data acquisition is forced due to the reliance of optical sensors on the sun’s electromagnetic radiation as a source of energy, although these sensors provide useful thermal data during night-time acquisitions as well [54]. Weather dependence of optical images is linked to the fact that the system uses short-wavelength electromagnetic energy that fails to penetrate dense atmospheric conditions common in hazy or cloudy skies [39]. Nevertheless, advances in image pre-processing continue to tackle these problems [56] to improve the signal-to-noise ratio vital for reliable information extraction about a target.

Traditional and advanced classification methods can be used to exploit spectral information of optical images for species discrimination. Traditional approaches employ the classical classification algorithms such as K-means and Iterative Self Organizing Data Analysis Technique (ISODATA) [57] Maximum Likelihood Classification (MLC) [58] and Minimum Distance-to-Means Classifier [59]. Applying ISODATA unsupervised classification using SPOT 5 and QuickBird in a Subtropical savanna (20 000 km^2^ area coverage), [60] for example discriminated between 19 woody plant species recorded from plots measuring 10 m in diameter. The study reported overall accuracy of 95%. Conspicuously, savanna environments are characterised by patches (clusters of woody plants) that vary in size, necessitating multi-scale analysis [4]. To assess the degree of information lost when using medium to high spatial resolution images that capture species coverage at varied scales, [61] classified eight plant species from 15-m plots in Montane savanna covering 20 000 km^2^. The authors utilised ISODATA classification and four images including Landsat, IKONOS, QuickBird and Worldview-2. The classification resulted in overall accuracies ranging between 75%– 91%. The aforementioned classification algorithms use hard techniques that allocate each image pixel to one class [62]. In contrast, soft techniques allocate each pixel to more than one class by applying membership weighting for each class. Adopting this approach, [63] compared fuzzy classification (soft technique) and Maximum Likelihood (ML) (hard technique) to classify six species recorded from plots measuring 30-m^2^ in a Sub-tropical savanna (456 498 ha). Overall classification results showed higher performance for fuzzy classification (87%) compared to ML classification (77%). Overall, traditional algorithms for classification of optical image suffer from data distributional assumptions and data input restrictions [54, 62].

Advanced classification methods including machine-learning and deep-learning algorithms do not make assumptions about data distribution and can model complex class signatures [64]. These approaches handle samples with a large number of variables while minimising error during the classification process [65]. Most notable advanced classification methods that have wide acceptance in remote sensing of species classification include Random Forest (RF) [66], Support Vector Machine (SVM) [67], Decision Trees (DT) [68], Boosted Decision Trees [69], Artificial Neural Network (ANN) [59], Deep Neural Network (DNN) [70] and Convolutional Neural Network (CNN) [71]. [65] implemented RF and SVM algorithms to Worldview-3 and discriminated seven woody plant species recorded from 14 m^2^ plots in a Sub-tropical savanna covering 247 km^2^. The authors found overall accuracies of 83% (RF) and 88% (SVM). Similarly, [72] discriminated one invasive species type from coexisting species and land cover types using SVM classification, Landsat-8 and SPOT-6 images in a Montane savanna (1 660 km^2^). The study trained the spectra of each imagery on samples collected from 5 m^2^ plots. They reported overall accuracies of 83% (Landsat-8) and 86% (SPOT-6). [73] used RF and Worldview-2 for a bi-seasonal analysis of seven woody plants. The authors recorded woody plant species from 4-m^2^ plots in a Subtropical savanna forest covering 71 km^2^ and reported overall accuracy of 86%.

Complex savanna environments present challenges to the effective separation of woody plant species that may have similar spectral signatures. [74] proposed a deep learning classification framework “Diverse Region-Based CNN” with more discriminative power for extracting spatial-spectral features than conventional machine learning algorithms. The approach is based on the assumption that adjacent pixels often consist of similar features and can represent the same class as the focal pixel, and therefore the spatial information of pixels should be factored in to determine class assignment. Testing the approach in a complex Tropical savanna region covering 325 ha, [64] utilised CNN on aerial photographs to classify nine woody plant species. Using a Global Position System (GPS), 370 plant species were identified in the field and manually delineated from the aerial photographs. The CNN approach followed the majority voting rule to identify tree crowns giving it the advantage of being more straightforward and faster compared to other machine learning algorithms. The authors reported an overall classification accuracy of 98%. [75] utilised optical image derived from Unmanned Aerial Systems (UAS) and three CNN approaches including FasterRCNN, RetinaNet and YOLOv3 to separate *Dipteryx alata* species from coexisting species and land cover types in a Tropical savanna area covering 150 000 m^2^. The authors reported overall classification accuracies ranging between 82% to 93%, with RetinaNet achieving the best results. The above literature has shown the efficacy of optical remote sensing data combined with a multiple classification algorithms to map and monitor species diversity in savanna ecosystems. Table 1 provides a list of selected studies that categorically classified woody plant species in the savanna environment using optical remote sensing.

**Table 1:** Examples of studies that categorically classified woody plant species in savanna using optical remote sensing.

Savana Biome Ecoregion	Number of species	Area covered	Sensor (Platform)	Spatial Resolution (m)	Categorical-scale parameter	Classification method	Results	Reference
Montane	10 species	6 000 ha	WorldView-2 (Spaceborne)	0.5	Individual tree crowns	Support Vector Machine (SVM)	Overall Accuracy = 89	[76]
Mediterranean	17 species	70 ha	Hyperspectral (Airborne)	0.56	Class separability	SVM, Artificial Neural Network (ANN) and K-Nearest Neighbour (K-NN)	Overall Accuracy (75%–84%)	[77]
Tropical-Subtropical	40 species	210 ha	QuickBird (Spaceborne)	0.65	Individual tree crowns	Maximum Likelihood Classifier (MLC)	Kappa coefficient (0.58–0.99)	[78]
Tropical	Two species and two land cover types	5 km^2^	QuickBird (Spaceborne)	0.65	Class separability	MLC	Overall Accuracy (60%–73%)	[79]
Montane	Four species	265 km^2^	Worldview-2 and IKONOS (Spaceborne)	0.5 0.8	Class separability	Random Forest (RF)	Overall Accuracy (67%–76%)	[80]
Tropical	Eight species	251.8 ha	ProSpecTIR-VS system (SpecTIR, Inc., USA) Hyperspectral (Airborne)	1	Individual tree crowns	Linear Discriminant Analysis (LDA) and SVM	Overall Accuracy (57%–85%)	[81]
Tropical-Subtropical	24 species	1 210km^2^	Sentinel-2A (Spaceborne)	10	Class separability	SVM, Nearest Neighbour (NN), RF and Classification Trees	Overall Accuracy (50%–74%)	[82]
Montane	One species and two land cover types	1 660km^2^	SPOT-5 and Landsat-7 (Spaceborne)	5 30	Class separability	SVM	Overall Accuracy (83%–86%)	[72]
Tropical-Subtropical	Four species and three land cover types	580 km^2^	Sentinel-2 and Landsat-8 (Spaceborne)	10 30	Class separability	RF and SVM	Overall Accuracy (90%–93%)	[83]
Tropical-Subtropical	22 species	530 km^2^	SPOT-5 and Landsat-5 (Spaceborne)	5 30	Class separability	MLC	Overall Accuracy (30%–53%)	[84]
Montane and Tropical	Six species and three land cover types	315 ha	Landsat-8 (Spaceborne)	30	Class separability	ANN, Decision Tree (DT), SVM and RF	Overall Accuracy (48%–73%)	[85]

### 4.2 Categorical classification of savanna woody plant species using RADAR remote sensing

RADAR remote sensing system operates in the microwave region (1 mm to 1 m) of the electromagnetic spectrum; as a result, it is capable of passing through cloud cover, haze, as well as foliage to detect understory features [86, 87]. A key advantage of active RADAR systems over optical remote sensing is that they provide own source of electromagnetic energy allowing them to be operated during day and night. Unlike in optical data, however, pixels in RADAR carry information from multiple scatterers that result in a highly complex data structure [88]. This phenomenon results in speckled appearance that require careful interpretation, especially in classification applications [89]. In addition, the topography is also a major limitation in mountainous regions due to geometric and radiometric effects when data are mapped to ground-range images; however, common public RADAR data (e.g., Sentinel-1) offer pre-processed product ready for application purposes [90].

Both traditional and advanced classification methods have been applied to RADAR data for categorical-scale species discrimination in savanna environments. For example, [91] used Wishart unsupervised classification and RADARSAT-2 data to discriminate between different species (n = 19) recorded in plots measuring 5 m x 8 m in a Tropical savanna (462 km^2^). The study reported overall accuracy of 62% and also found the importance of incidence angle (a characteristic of RADAR acquisition mode) on classification accuracy with images taken at low incidence angle performing better. [92] classified (overall accuracy = 86%) five plant species recorded from 25-m^2^ plots in a Subtropical savanna (11 ha) using Wishart classification applied to L-band Polarimetric Synthetic Aperture Radar (PolSAR). [93] applied ML classification to L-band Phased Array type L-band Synthetic Aperture Radar (PALSAR) in a Tropical savanna (area of 125 × 100 km^2^) to discriminate between three species and coexisting land cover types recorded from 12.5-m^2^ plots. The study reported overall accuracy of 87%. [94] combined fuzzy ML classification and TerraSAR-X, RADAR data to discriminate between three species and two land cover types in a Tropical savanna covering 354.6 ha. Plant species were recorded using Unmanned Aerial System (UAS) within plots measuring 0.2-m^2^, and the study found an overall classification accuracy of 89%.

Machine learning algorithms have been explored to improve savanna plant species discrimination using RADAR data. For instance, [95] applied DT classification (overall accuracy = 52%) to Sentinel-1 C-band data classifying eight woody plant species recorded from 3-m^2^ plots in a 2 550 km^2^ Tropical savanna. They also compared different polarisation (orientation modes of RADAR images) including Vertical-Vertical (VV), Vertical-Horizontal (VH) and VV/VH images and found better results from VH cross polarisation. Using PALSAR and RF, [96] classified (overall accuracy = 72%) three species and coexisting land cover types recorded from 30 m x 30 m plots in a Subtropical savanna measuring 3 697 km^2^. [97] classified three woody plant species derived from plots measuring 10 m radius in a Tropical savanna region covering 224 300 km^2^. They applied RF and Multilayer Perceptron (MLP) classifiers to Sentinel-1 C-band image and found accuracies of 75% and 83%, respectively. Unlike in optical remote sensing, the use of deep learning algorithms with RADAR data for the purpose of classifying savanna plant species is limited although these algorithms hold a promise for complex data structures offered by RADAR [98]. [99], for example, classified three plant species and coexisting land cover types in a Mediterranean Subtropical savanna environment covering 400 km^2^. They specifically applied Recurrent Neural Network (RNN) on Sentinel-1 C-band image and found an overall accuracy of 90%. Table 2 provides examples of studies that used RADAR remote sensing for categorical classification of woody plant species in different savanna environments.

**Table 2:** Examples of studies that categorically classified woody plant species in savanna using RADAR remote sensing.

Savana Biome Ecoregion	Number of species	Area covered	Sensor (Platform)	Spatial resolution (m)	Categorical-scale parameter	Classification method	Results	Reference
Tropical	Four species and two land cover types	300 ha	PoLSAR (Spaceborne)	8	Class separability	RF	Overall accuracy (50%–83%)	[100]
Tropical	Two species and four land cover types	40 km^2^	PoLSAR and RADARSAT-2 (Spaceborne)	8 10	Individual tree crowns	RF	Overall accuracy (75%–83%)	[34]
Tropical	One species and three land cover types	27 km^2^	Sentinel-1 C-band (Spaceborne)	10	Class separability	Pixel Based and Object-Based classification	Overall accuracy (58%–85%)	[101]
Tropical	Eight species and three land cover types	25 520 km^2^	Sentinel-1 C-band (Spaceborne)	10	Class separability	DT	Overall accuracy = 52%	[95]
Tropical	Six species and three land cover types	139 km x 71 km	RADARSAT-2 (Spaceborne)	10	Class separability	K-mean unsupervised	Overall accuracy = 86%	[102]
Tropical	Nine species and one land cover type	160 000 km^2^	RADARSAT-2 and ALOS PALSAR (Spaceborne)	10 25	Class separability	Object Based classification	Overall accuracy = 80%	[32]
Montane	Three species and four land cover types	1 314 km^2^	RADARSAT-2 and ALOS PALSAR (Spaceborne)	10 25	Class separability	Wishart supervised classification	Overall accuracy = 86%	[103]
Tropical	Four species and Land cover types	3 660 km^2^	RADARSAT-2 and ALOS PALSAR (Spaceborne)	10 25	Class separability	Naive Bayes (NB), DT, RF, Multilayer Perceptron (MP) and SVM	Overall accuracy (63%–74%)	[104]
Tropical	Two species and three land cover types	11 240 km^2^	ALOS PALSAR (Spaceborne)	25	Class separability	Object Based classification	Overall accuracy = 87%	[105]
Tropical	Five species and two land cover types	30 km x 60 km	ALOS PALSAR (Spaceborne)	25	Class separability	SVM	Overall accuracy = 85%	[106]
Tropical	One species one land cover	68 000 km^2^	ENVISAT ASA and ALOS PALSAR (Spaceborne)	25 25	Class separability	MLC	Overall accuracy = 87%	[107]

### 4.3 Categorical classification of savanna plant species using LiDAR remote sensing

LiDAR remote sensing captures information about species in three dimensions: latitude, longitude and altitude [54]. It is therefore suitable for structural assessment of vegetation such as canopy cover, height and volume [108, 109]. LiDAR systems scan ground features using laser points at high sampling rates and relatively small laser footprint size, and are therefore able to even penetrate through small canopy gaps [110]. This effect provides the capability for detailed geometrical reconstruction of different species. Because of its active illumination mode, LiDAR can be operated during day and night, and is not constrained by weather conditions that may block the sun’s energy. Furthermore, the (near) nadir looking approach used by the system makes LiDAR to be largely unaffected by geometrical distortions of landscapes [111]. Though LiDAR is desirable for producing accurate results, it is expensive to apply it to large spatial areas. Moreover, like hyperspectral remote sensing, LiDAR systems collect a large volume of data that require high performance computers for analysis. As in the case of optical and RADAR data, LiDAR data have been used with traditional and advanced classification methods to categorically classify wood species in savanna ecosystems. [112] for example, applied ML classification to LiDAR height metrics classifying 41 woody plant species identified from 4-m^2^ plot sizes in a Subtropical savanna region covering 400 ha. The study reported overall accuracy of 81%. [113] used K-means unsupervised classification algorithm on LiDAR data in a Mediterranean savanna (16.5 ha) to classify five species recorded in 7 m x 7 m plots. Height metrics derived from LiDAR data resulted in an overall accuracy of 97%.

Given the large amount of structure- and intensity-related information (metrics) that can be extracted from LiDAR point clouds, traditional classification approaches may offer suboptimal accuracy. It is therefore logical to exploit machine learning algorithms that handle such large data. [114] used small-footprint LiDAR data to distinguish between five woody plant species recorded from plots measuring ~11 m in radius within a Temperate savanna region (405 m^2^). RF algorithm applied to 34 LiDAR tree height metrics resulted in an overall accuracy of 95%. Despite the success, the study also noted that there was a significant reduction in the number of LiDAR pulses that reach the forest understory in closed canopy forests, inhibiting the characterisation of understory species. In order to account for understory foliage in a Temperate savanna region covering 2 km^2^, [111] classified three species using small-footprint LiDAR and tree crown size data in plots measuring 20 m^2^. The authors used linear unmixing and RF classification algorithms that returned overall accuracies of 81% and 84%, respectively. Furthermore, it is worth noting that different seasons exhibit unique characteristics which ultimately influence classification of LiDAR remote sensing similar to optical remote sensing. [115] compared woody species classification accuracies in leaf-on (wet season) and leaf-off (dry season) conditions in Temperate savanna region (100-m^2^) using terrestrial LiDAR data. The study used RF classification and LiDAR-derived height metrics, achieving overall accuracies of 77% for leaf-off and 78% for leaf-on conditions, indicating the importance of season for species discrimination.

A key advantage of LiDAR data is that it offers high level of detail for individual tree mapping. In a Tropical-Subtropical savanna [116] applied voxel-based tree extraction and noise removal approach to identify four species in study area A (1 ha) and eight species in study area B (1 ha) using LiDAR and Deep Belief Network (DBN). Tree crowns were subsequently rasterised and classified using DBN model at an overall accuracy of 93% and 95% for two study areas respectively. Similarly, [117] classified 50 000 individual tree samples (tree crowns) belonging to 10 species along a 4 km road in a Montane savanna region using DBN model and LiDAR-derived voxels (volumetric structures) achieving an overall accuracy of 86%. In a Temperate savanna covering 7 440 ha, [118] classified woody plant species (n = 11) from LiDAR-derived individual tree crowns and using CNN (overall accuracy = 81%). Table 3 presents examples of additional studies which applied LiDAR data and categorical classification to assess woody plant species diversity in different savanna regions.

**Table 3:** Examples of studies which categorically classified woody plant species in savanna using LiDAR remote sensing.

Savana Biome Ecoregion	Number of species	Area covered	Sensor (Platform)	Categorical-scale parameter	Classification method	Results	Reference
Montane	Three species	100 ha	Small-footprint 0.1 points/m^2^ (Airborne)	Individual tree crowns	RF	Overall Accuracy = 64%	[119]
Temperate	11 species	7 440ha	Small-footprint 0.13 points/m^2^ (Airborne)	Individual tree crowns	CNN	Overall Accuracy (65%–90%)	[118]
Temperate	40 species	316 km^2^	Small-foot-print 3.5 points/m^2^ (Airborne)	Individual tree crowns	Gaussian fuzzy membership	Overall Accuracy = 69%	[120]
Tropical	Five species	300 km^2^	Waveform (Airborne)	Plant Area Index	RF	Overall Accuracy = 81%	[121]
Temperate	Five species	10 km^2^	Waveform (Airborne)	Leaf Area Index	RF	Overall Accuracy = 89%	[122]
Montane	Six species	1 103 ha	Small-footprint and Full-Waveform (Airborne)	Individual tree crowns	RF	Overall Accuracy (69%–86%)	[123]
Mediterranean	24 species	6 km^2^	Waveform (Airborne)	Individual tree crowns	DT RF	Overall Accuracy = 79%	[124]
Montane	10 species	250 m x 500 m	Waveform (Airborne)	Individual tree crowns	Deep Boltzmann Machines (DBM)	Overall Accuracy = 86%	[117]
Tropical-Subtropical	One species and two land cover types	50 m x 80 m	Waveform (Airborne)	Individual tree crowns	DT	Overall Accuracy = 69%	[125]

## 5. Remote sensing of savanna woody plant species diversity using continuous-scale assessment

### 5.1 Continuous-scale assessment of savanna plant species using optical remote sensing

Categorical classification of vegetation types suffers from misallocation of species to incorrect classes. Continuous-scale species diversity assessment overcomes this by building statistical relationships between diversity indices and remotely-sensed data. This bodes well in biodiversity assessment that may seek to focus on Species richness measures of a given area. Continuous-scale assessment benefits greatly from spectral data provided by remote sensing systems. For example, [126] estimated three diversity indices including Species richness, Simpson and Shannon Wiener indices derived from 20-m^2^ plots in a Temperate savanna (113 700 ha). They applied Spearman correlation test to assess the relationship between diversity indices with Difference Vegetation Index (DVI), Normalised Difference Vegetation Index (NDVI), Enhanced Vegetation Index (EVI), Vegetation Index (VI) and Greenness Ratio derived from Landsat 8 data. Species richness was negatively correlated (r = -0.18) with DVI and strongly and positively correlated (r = 0.59) with NDVI. Similarly, Shannon diversity index showed a negative correlation (r = -0.12) with DVI and positive correlation (r = 0.60) with NDVI. Almost comparable results were recorded for Simpson index. In a Subtropical savanna area covering 100 km^2^, [127] derived Shannon and Simpson diversity indices from plots measuring 15 m^2^ and regressed the indices against NDVI metrics extracted from both Landsat 8 and Worldview-2 datasets. The study reported coefficients of determination (R^2^) of 0.32 for Shannon–Landsat-8 NDVI, 0.72 for Shannon–Worldview-2 NDVI, 0.58 for Simpson–Landsat-8 NDVI and 0.69 for Simpson–Worldview-2 NDVI.

Reliance on spectral properties data alone may not fully capture species variability in a given area because identical spectral reflectance values can correspond to unique species [128]. This shortcoming can be mitigated by using texture information that uses the spatial arrangement of pixels in an image [129]. Applying this principle, [130] derived Shannon diversity index from 30 x 30 m plots in a Temperate savanna covering 24 281 ha. They, then, used linear regression to assess the relationship between the diversity index and eight Grey Level Co-occurrence Matrices (GLCM) extracted from Landsat-7 achieving R^2^ = 0.01–0.60. [48] estimated woody plant species diversity in Montane savanna area (651 ha), dominated by morphologically similar woody plant species. The study applied the all-subsets regression to correlate Shannon diversity index quantified from field inventory of 15 m radius plots with GLCM derived from individual bands of WorldView-2 imagery. The authors reported adjusted R^2^ = 0.41–0.46 and Root Mean Square Error (RMSE) = 0.60–0.58. [131] derived empirical relationships of GLCM, Leaf Area Index (LAI) with Shannon diversity index calculated surveys of plots measuring 20 m^2^ in a Montane savanna area (258 km^2^). Linear regression analysis was used to correlate the GLCM, LAI and Shannon diversity index with adjusted R^2^ values ranging between 0.73 and 0.74.

While the above reviews prove the importance of spectral and textural information for species diversity assessment individually, the assessment can benefit by combining the two types of information. For example, [132] derived Shannon, Simpson and Species richness indices from 50 plots measuring 90 m x 90 m in a Montane savanna. These indices were regressed against eight individual bands, four spectral indices and three GLCMs extracted from Landsat-8 achieving R^2^ of 0.36–0.41. [133] correlated eight GLCMs and two vegetation indices derived from QuickBird with Species Richness (R^2^ = 0.44–0.59), Shannon-Wiener diversity index (R^2^ = 0.46–0.6), and Simpson’s diversity index (R^2^ = 0.42–0.57) for woody plant species in Tropical environment (20 x 5m plots) covering 12.6 km^2^. Overall, findings related to optical remote sensing studies indicate that it is preferable to assess species diversity with high spectral and spatial resolutions to identify subtle differences in plant species. Further examples of continuous-scale assessment of woody plant species in savanna are provided in Table 4.

**Table 4:** Examples of studies that quantified woody plant species diversity in the savanna using optical remote sensing and continuous-scale.

Savana Biome Ecoregion	Number of species	Area covered	Sensor (Platform)	Spatial resolution (m)	Continuous-scale parameter	Analysis methods	Results	Reference
Temperate	Five species	150 ha	Worldview-3 (Spaceborne)	0.4	NDVI	Exponential regression	R^2^ = 0.84	[134]
Montane	26 species	651 ha	Worldview-2 (Spaceborne)	0.5	GLCM	All-possible subsets regression	Adj R^2^ (0.41–0.46) RMSE (0.60–0.58)	[48]
Montane	Three species	258 km^2^	GF2 and SPOT-6 (Airborne and Spaceborne)	1 1.5	NDVI, Soil Adjusted Vegetation Index (SAVI), Enhanced Vegetation Index (EVI), Normalized difference index using green band (NDVIg), Chlorophyll index using green band (CIg) and GLCM	Random Forest regression model	R^2^ (0.78–0.92)	[135]
Montane	Eight species and three land cover types	23 404 ha	SPOT-6 (Spaceborne)	1.5	NDVI, Renormalized Difference Vegetation Index (RDVI), Ratio Vegetation Index (RVI), Difference Vegetation Index (DVI), Modified Simple Ratio (MSR), (EVI)	Random Forest regression model	R^2^ = 0.74	[136]
Temperate	Two species	40 ha	Rapid Eye and Landsat-7 (Spaceborne)	5 30	Normalized Difference Wetness Index (NDWI), NDVI and Normalized Difference Red Edge (NDRE),	Multiple linear regression	Adj R^2^ (0.9–0.92)	[137]
Mediterranean	Seven species	20 ha	Sentinel-2A and Landsat-8 (Spaceborne)	10 30	NDVI	Linear regression	R^2^ (0.42–0.74)	[138]
Temperate	27 species	113 700 ha	Landsat-8 (Spaceborne)	30	DVI, NDVI, EVI, Vegetation Index (VI) and Greenness Ratio	Spearman correlation	Species richness r = -0.18 (DVI) and r = 0.59 (NDVI). Shannon diversity r = -0.12 (DVI) and r = 0.60 (NDVI). Simpson index r = -0.11 (DVI) and r = 0.59 (NDVI)	[126]
Tropical	25 species	844 453 km^2^	Landsat-5, and Landsat-8 (Spaceborne)	30 30	Simple Ratio Index (SRI) NDVI, SAVI, EVI and Leaf Area Index (LAI)	Pearson correlations and Principal Components Analyses	r (0.34–0.744)	[139]
Tropical	Three species and three land cover types	22 270 km^2^	Moderate Resolution Imaging Spectroradiometer (MODIS) (Spaceborne)	250	NDVI, EVI, LAI, and Fraction of Photosynthetically Active Radiation (FPAR)	Multiyear Partial Least Square Regression	R^2^ (0.76–0.91)	[140]

### 5.2 Continuous-scale assessment of savanna woody plants using RADAR remote sensing

RADAR images provide backscatter related to intensity, amplitude and interferometry information acquired in different customisable acquisition modes including wavelengths, incidence angles and polarisations (orientations) of emitted and received radiations. The variation in interaction of backscatter depending on target characteristics further complicate the information content of RADAR data. Such complexity might render the data suitable for continuous-scale statistical assessment of species diversity [141]. Exploring the efficacy of image intensity for species diversity estimation, [142] used images of different polarisations including HV, (HH, VV, and VH derived from Sentinel-1 C-band and Advanced Land Observation System Phased Array L-band Synthetic Aperture Radar (ALOS PALSAR) for species diversity estimation in a Montane savanna (6 km^2^). Regression analysis relating the images with Shannon diversity index calculated from surveys of 10-m^2^ plots indicated good correlations for ALOS PALSAR data (HV polarisation R^2^ = 0.63; HH polarisation R^2^ = 0.58) compared to Sentinel-1 C-band (VV polarisation R^2^ = 0.054 and VH polarisation R^2^ = 0.044). [143] calculated Shannon diversity index of species surveyed from 10 m radius plots in a Montane savanna region covering 1752km^2^ and subsequently correlated it with Dual Polarisation SAR Vegetation Index (DPSVI) obtained from VV and VH polarisations of Sentinel-1 C-band. Simple linear regression analysis returned R^2^ ranging between 0.70 and 0.75, with DPSVI computed from VH polarisation performing better than VV polarisation. [144] utilised RADARSAT-2 to estimate Shannon diversity index calculated from 1 m^2^ plot sizes in a Montane savanna (260 000 ha). Simple linear regression results showed R^2^ = 0.66 for HH and R^2^ of 0.71 for HV. Earlier, [145] calculated Shannon diversity index from 30-m^2^ plots in Tropical savanna covering 60 km x 18 km and correlated the index four polarisations of AIRSAR data (HH, VV, HV and VH). The results showed R^2^ ranging between 0.04 for HV polarisation to R^2^ = 0.95 for HH polarisation.

Spatial associations of the scattering properties of target features can be extracted from RADAR through GLCMs for estimating plant species diversity [143]. [146] calculated Shannon diversity index from 15-m radius plots in a Tropical savanna measuring 200 km^2^. Simple linear regression analysis was used to compare the association of GLCMs extracted from Japanese Earth Resources Satellite 1 (JERS-1) SAR and Shannon diversity, recording R^2^ ranging between 0.04 (for contrast GLCM) and 0.85 (for entropy GLCM). In a Subtropical savanna covering 1 100 km^2^, [147] extracted eight GLCMs from RADARSAT-2 and correlated them with Shannon diversity index calculated from species in plots measuring 15 m in radius. Using multiple linear regression, the authors reported adjusted R^2^ ranging between 0.40 and 0.9. [148] also estimated plant species diversity recorded from plots measuring 25 m x 25 m in a Tropical savanna (25 ha). Eight GLCMs statistics were recorded from P band of TomoSAR data and using linear regression the best correlation between species diversity and GLCMs statistics resulted in R^2^ = 0.70.

A valuable extension of RADAR technology relates to interferometry which combines multiple images taken from different positions or at different times and quantifies their phase difference to identify similarity (coherence) and dissimilarity (incoherence) of features [54]. Height variations obtained using interferometric analysis can be used to discriminate between vegetation species. [149], for example, assessed the suitability of coherence data extracted from RADARSAT-2 for species diversity estimation in a Tropical savanna covering 85 km^2^. Linear regression analysis correlating the coherence data with plot-level field surveys resulted in an R^2^ of 0.5. Recently, [141] recorded tree height information to modelling species diversity (Shannon diversity index) from 5-m^2^ plots in a Montane savanna covering 1 102 km^2^. They, then, regressed the index against coherence data derived from ALOS/PALSAR imagery and found R^2^ of 0.67. Table 5 presents examples of additional studies which applied RADAR data and continuous-scale assessment of woody plant species in different savanna regions.

**Table 5:** Examples of studies that estimated savanna woody plant species diversity using RADAR remote sensing and continuous-scale.

Savana Biome Ecoregion	Number of species	Area covered	Sensor (Platform)	Spatial Resolution (m)	Continuous-scale parameter	Method	Results	Reference
Flooded	Four species and one land cover type	153 500 ha	RADARSAT-2 (Spaceborne)	10	HH+HV, VH+VV polarisations	Univariate and multivariate linear regression	R^2^ = 0.48	[150]
Tropical	Five species	1 011 ha	TanDEM-X (Spaceborne)	12	HH and VV polarisations	Regression analysis	r = 0.93	[151]
Temperate	11 species	1 620 m^2^	Sentinel-1 C-band and PALSAR-2 (Spaceborne)	10 x25	VH and VV polarisations	Multiple linear regression	R^2^ (0.56–0.93)	[152]
Montane	67 species	6.25 km^2^	Sentinel-1 C-band ALOS PALSAR L band (Spaceborne)	10 x25	VH and VV polarisations	Regression analysis	R^2 ^(0.04–0.66)	[142]
Tropical	Seven species and three land cover types	15 000 km^2^	JERS-1 and ALOS PALSAR (Spaceborne)	12.5 x 25	HH and HV polarisations	Linear regression	R^2^ (0.86–0.95)	[153]
Montane	11 species	684 km^2^	ALOS PALSAR (Spaceborne)	25	HH and HV, HH + HV, HH − HV and HH/HV polarisations	Multiple linear regression	R^2^ (0.53–0.55)	[154]
Tropical	Three species	1 750 km^2^	ALOS PALSAR (Spaceborne)	25	HH and HV Polarisations	Linear regression	R^2^ (0.53–0.70)	[155]
Montane	Two species	9 km^2^	ALOS PALSAR (Spaceborne)	25	HH and HV polarisations	Linear regression	R^2^ (0.56–0.93)	[156]
Flooded	Three species	181 037 km^2^	ALOS PALSAR (Spaceborne)	25	HV and HH/HV polarisations	Regression analysis	R^2^ (0.67–0.95)	[157]
Montane	Three species	308 245 km^2^	ALOSPALSAR (Spaceborne)	25	HH and HV polarisations	Regression analysis	R^2^ = 0.59	[158]

### 5.3 Continuous-scale assessment of savanna woody plants using LiDAR remote sensing

Continuous-scale species diversity assessment using LiDAR remote sensing can be applied at plot level and individual tree level given the high spatial detail afforded by the system. Although savanna environments exhibit discontinuous tree species distribution, which tends to affect the applicability of plot level analysis, the approach has largely achieved good results. In general, plot level approaches affords better correlations with large plot sizes combined with LiDAR data that provide high pulse density capable of capturing sufficient number of tree crowns. For example, [159] estimated Shannon diversity index from surveys in 400 m^2^, 1000 m^2^ and 2200 m^2^ plots in a Tropical savanna covering 9 km^2^. They correlated the index with LiDAR derived canopy density and tree height metrics (mean and standard deviation) using Ordinary Least Squares. Low associations between field and LiDAR data were recorded from 400-m^2^ and 1000-m^2^ plots (R^2^ 0.18 and 0.19, respectively), compared to R^2^ = 0.49 for 2 200-m^2^ plot. Using rather small plot sizes, [160] successfully correlated (r = 0.85) Shannon diversity index and LiDAR-derived tree height in 10-m radius plots covering a total of 97 km^2^ study area in a Mediterranean savanna region. In addition to height metrics extraction, canopy cover is also used for plot level species diversity prediction using LiDAR remote sensing. For instance, [161] calculated modified Shannon–Wiener and Evenness indices extracted from plots measuring 2 and 4 m radius in a Subtropical savanna (1600 ha). These diversity indices were correlated to canopy cover and height using linear regression recording R^2^ = 0.72–0.82.

Although overstorey and understorey LiDAR returns can be successfully separated in sparsely vegetated savanna environments, the separation capabilities becomes less effective in canopies with dense and continuous architecture especially when LiDAR point density is low [160, 162]. Canopy height models (CHM), which simulate continuous surfaces (grids) of canopy tops, have been used to solve such problem instead of directly relying on point cloud height information. For example, [162] used LiDAR metrics generated from CHM to correlate Rao’s Q and Shannon diversity indices extracted from 100-m^2^ plots in a Mediterranean savanna of 270 ha. Using simple linear regression, the authors reported R^2^ of 0.73 for Shannon diversity index and R^2^ of 0.75 for Rao’s Q. [138] related Simpson diversity index (R^2^ = 0.56) and Shannon diversity index (R^2^ = 0.63) calculated from 100-m^2^ plots with CHM derived from LiDAR in a Temperate region (270 ha). In a Mediterranean environment (20 000 ha), [163] measured Shannon and Simpson diversity indices from 400- and 2840-m^2^ plots. Correlation of the diversity indices and LiDAR derived CHM resulted in adjusted R^2^ of 0.63 and 0.89 for Shannon diversity index and Simpson diversity index, respectively.

The aforementioned area-based (plot level) analysis suffer from overfitting, thus individual tree based approaches are more suited for heterogeneous savanna environments [109, 116]. Individual tree based approaches involve locating tree canopies first, provided that there is sufficient point density data. It is critical to have a reasonable window size when delineating individual tree crowns. Large window sizes can capture multiple trees in dense environments while a smaller window creates more trees than the actual available trees. For example, [164] underestimated available plant species when using smaller window sizes for individual tree crown delineation in a 50 ha Tropical environment. In contrast, a study by [165] in a Mediterranean environment overestimated functional beta diversity attributed to large window sizes used to identify individual crowns. In a Mediterranean savanna covering 1295 ha, [116] correlated Species richness index calculated from individual trees counted in 5 m^2^ plot sizes. Tree crowns were segmented from a Digital Canopy Model (DCM) derived from LiDAR data were able to estimate the index with R^2^ = 0.64. [166] utilised cluster analysis to segment individual trees (650) delineated from plots measuring 3 m x 3 m in a Temperate savanna covering 35 ha. The study used simple linear regression to relate LiDAR derived crown segments with Species richness (R^2^ = 0.76) and Shannon diversity index (R^2^ = 0.84). Theoretically, individual tree based approaches can be surrogates of fieldwork thus, minimising manual field work. Further examples of studies that applied LiDAR remote sensing to savanna woody plant species diversity assessment using continuous-scale analysis are provided in Table 6.

**Table 6:** Examples of studies that estimated savanna woody plant species diversity using continuous-scale and LiDAR remote sensing.

Savana Biome Ecoregion	Number of species	Area covered	Sensor (Platform)	Continuous-scale parameter	Method	Results	Reference
Montane	Three species	50 ha	Small-footprint 0.01–0.02 points/m^2^ (Airborne)	Individual tree crowns	Linear regression	R^2^ = 0.80	[167]
Tropical-Subtropical	Three species	25 km^2^	Small footprint 0.02 points/m^2^ (Airborne)	Individual trees crowns	Non-linear regression	R^2^ (0.83–0.86)	[168]
Temperate	Three species	2 601 ha	Small-footprint 0.19 points/m^2^ (Airborne)	Forest strata	Random Forest model	R^2^ (0.72–0.88) RMSE = 0.06%	[169]
Montane	Six species	1 103 ha	Small-footprint 0.25 points/m^2^ (Airborne)	Individual trees crowns	Regression Analysis	Adj-R^2^ (0.50–0.93)	[123]
Temperate	Six species	2 258 ha	Small-footprint 0.25 points/m^2^ (Airborne)	Individual trees crowns	Stepwise multiple linear regression	R^2^ = 0.8	[170]
Montane	One species and one land cover type	63 000 ha	Small-footprint 1.5 points/m^2^ (Airborne)	Tree height	Multiple linear regression	R^2^ = 0.71 RMSE = 34.84%	[171]
Mediterranean	One species	4.5 ha	Small-footprint 1.5 points/m^2^ (Airborne)	Individual trees crowns	Linear regression	R^2^ = 0.95	[172]
Tropical-Subtropical	Two species	3 300 ha	Small-footprint full-waveform 9 points/m^2^ (Airborne)	Leaf Area Index	Regression Analysis	R^2^ = 0.75	[173]
Montane	Four species	13 650 ha	Waveform (Airborne)	Plot level canopy height	Regression Analysis	R^2^ (0.73–0.89)	[174]
Tropical	Six species	50 m^2^	Small-footprint full-waveform 9 points/m^2^ (Airborne	Individual trees crowns, Leaf Area Index	Linear regression	R^2^ = 0.81	[175]
Tropical	Seven species	515 ha	Waveform (Airborne)	Leaf Area Index	Regression Analysis	R^2^ = 0.42 RMSE = 1.91	[176]
Montane	Three species	130 000 km^2^	Waveform (Airborne)	Individual trees crowns	Linear regression	r = 0.91	[177]

## 6. Multi-source remote sensing for savanna woody plant species diversity assessment

### 6.1 Multi-source remote sensing

Advances in technology, coupled with an impetus in space exploration has seen a plethora of remotely sensed data available to the remote sensing community, providing more opportunities for multi-sensor data integration [89, 135, 178–181]. To take advantage of this, data fusion approaches have been applied for both categorical and continuous-scale species diversity estimation. Data fusion is a technique of integrating data obtained from a single sensor or multiple sensors to produce better information content than offered by individual dataset [89, 182]. Generally, data fusion is implemented at three different levels including pixel, feature and decision level [29, 183]. Pixel-level fusion combines raw data (mainly optical images) from multiple sources into single resolution data (spatial–spectral). Feature-level fusion operates at higher processing levels and extracts features from different data sources and then combines them into one or more feature maps that may be used instead of the original data for further analysis. Decision level fusion represents the highest level of the three data fusion approaches and uses knowledge-based procedures to combines the results from various algorithms [184, 185].

### 6.2 Categorical classification of savanna woody plants using multi-source remote sensing

Categorical classification of savanna species using data fusion can exploit both similar (e.g., optical—optical) and different (optical—RADAR, optical—LiDAR and LiDAR—RADAR) datasets. [186] classified six plant species recorded from 30-m^2^ plots in a Subtropical environment (1610 km^2^) using Moderate Resolution Imaging Spectroradiometer (MODIS), Landsat-8 and HJ-Hyperspectral images. The study reported overall accuracies of 69% for Landsat-8, 70% for hyperspectral image, 79% for MODIS and 84% for the fused image. In a Tropical savanna covering 252 ha, [81] classified eight species recorded from 3 m x 3 m plots. Using data integrated from Worldview-3 and Hyperspectral images, the study reported overall accuracies of 79% (Wordview-3), 85% (Hyperspectral) and 89% for the fused image. Accessibility of optical images at no cost (such as Landsat and Sentinel), and improved accuracies due to enhanced spatial and spectral information perpetuated multi-sensor fusion of optical images for biodiversity assessment. However, an amalgamation of optical images suffers from colour distortion and spatial artifacts [178]. Analysts should therefore take into consideration these shortcomings when fusing optical data.

Perhaps, the most notable limitation of optical data is their inability in cloudy or hazy weather conditions making them inefficient for all year round monitoring of species diversity. Taking advantage of all-weather utility afforded by RADAR, [187] fused Sentinel-1 C-band and ALOS-2/PALSAR to classify two plant species and coexisting land cover types recorded from 12-m^2^ plots in a Mediterranean environment (28 km^2^). Classification results showed overall accuracies of 79% for Sentinel-1 C-band, 81% for ALOS-2/PALSAR and 89% for the fused image. Integrating the sensitivity of optical data to biochemical variations of plant species, and the sensitivity of RADAR backscattering to vegetation structure can maximize information extracted about vegetation characteristics and thus improve species discrimination [188, 189]. [190], for example, combined Sentinel-1 C-band with Sentinel-2 and Landsat-8 to classify two woody species in plots of varying sizes (15 m x 15 m and 50 x 50 m) in a Montane savanna region (242 813 ha). The classification resulted in overall accuracies of 65% for Landsat-8, 67% for Sentinel-2 and 76% for the fused image. Combining RADAR and hyperspectral data holds great capacity given the ability of the latter in mapping species diversity better than multispectral data. [191] for example, fused hyperspectral data acquired using Compact Airborne Spectrographic Imager (CASI) and RADAR data obtained using L-band AIRSAR in a Tropical savanna (50 km^2^) to classify nine plant species recorded from 2.5-m^2^ plots. They applied ML, ANN, Hierarchical ANN algorithms and found overall classification accuracies ranging between 58% and 80%, with the fused image providing the best result.

Similarly, combining optical and LiDAR data allows for exploitation of biochemical and structural information that can be used to classify species types. For example, [192] integrated LiDAR data and WorldView-2 image to classify eight woody plant species in a Tropical savanna covering 523 ha. Applying Dense Convolutional Network, SVM and RF, the analyses resulted in overall classification accuracies ranging between 52% and 83%. Combining the strength of hyperspectral and LiDAR is expected to identify detailed tree species diversity even in areas covered with morphologically similar plant species. In a Montane savanna covering 360 km x 70 km, [193] fused LiDAR data and hyperspectral data obtained from CASI image to classify 15 plant species recorded from 30-m^2^ plots and reported overall classification accuracies of 65% (hyperspectral image), 71% (LiDAR) and 76% for the fused image.

### 6.3 Continuous-scale assessment of savanna woody plants using multi-source remote sensing

Recent improvements in remote sensing technology associated with enhanced spatial and spectral resolution allows for improved species recognition and is particularly useful for continuous-scale species diversity estimation. [194] fused MODIS (coarse resolution) and RapidEye (high spatial resolution) in a Montane savanna covering 2915 km^2^. Shannon diversity index computed from 30 m x 30 m plots was then correlated with MODIS (R^2^ = 0.36), RapidEye (R^2^ = 0.45) and fused data (R^2^ = 0.71). Using better spatial resolution optical images (SPOT-6 and Gaofen-2 (GF2)) for data fusion in a Subtropical savanna covering 7 600 ha, [135] estimated Shannon diversity index from plots measuring 20 m x 20 m. Regression results from the study recorded R^2^ = 0.45 for GF2, R^2^ = 0.67 for SPOT-6 and better accuracy (R^2^ = 0.78) for the combined image. [195] combined Landsat-8 and Sentinel-2 images to quantify Species richness in 30-m^2^ plots in a Mediterranean savanna (12 000 km^2^). A simple linear regression analysis resulted in R^2^ = 0.86 (Landsat-8), R^2^ = 0.88 (Sentinel-2) and R^2^ = 0.98 for the fused image.

Combining LiDAR and optical data has become a popular method in the assessment of species diversity especially for high-spatial resolution end-products. [35] fused height and tree crown metrics extracted from LiDAR data with spectral information extracted from RapidEye to quantify the species complexity of a tropical savanna region covering 9 km^2^. That study used all-possible subset regression to relate Species richness and Shannon diversity indices recorded from 18-m radius plots, reporting R^2^ = 0.68 for RapidEye, R^2^ = 0.77 for LiDAR and R^2^ = 0.87 for the fused image. [196] calculated Shannon diversity index calculated from 18-m radius plots in a Tropical-Montane savanna environment covering 200 ha correlated them with LiDAR-derived canopy cover metrics and Landsat-8 spectral data. The correlation using linear regression analysis resulted in R^2^ = 0.55 for Landsat-8, R^2^ = 0.59 for LiDAR and R^2^ = 0.66 for the fused image. [197] combined LiDAR and Airborne Visible Infrared Imaging Spectrometer (AVIRIS) to estimate Species richness in 15-m radius plots in a Mediterranean environment (22 000 ha). Regression results from the study showed R^2^ = 0.69 (AVRIS), R^2^ = 0.77 (LiDAR) and R^2^ = 0.84 for the fused image.

Although commonly suitable to extract structural information, RADAR and LiDAR have also been combined to estimate woody plant species diversity. Crucially, LiDAR and RADAR data integration is useful for routine assessment of woody plant species diversity, since the sensors can be operated at all-weather conditions. It is therefore important to highlight few examples that exploited the combination of the two datasets. [198] fused TanDEM-X RADAR data and LiDAR data for estimating Species richness calculated from 20-m radius plots in a Temperate savanna region (3 100 ha). The estimation showed R^2^ = 0.39 for TanDEM-X, R^2^ = 0.51 for LiDAR and R^2^ = 0.71 for the fused image. Similarly, [199] combined TanDEM-X and LiDAR datasets and regressed against Species richness computed from 55-m^2^ plots in Tropical savanna with R^2^ = 0.76 for TanDEM-X, R^2^ = 0.78 for LiDAR and R^2^ = 0.83 for the fused image. [200] integrated ONERA’s SETHI airborne SAR data and LiDAR data in a Tropical savanna region covering 4910 km^2^. They specifically estimated Shannon diversity index obtained from 50 m × 50 m plots using the fused data that returned R^2^ = 0.80, compared to R^2^ = 0.67 for ONERA’s SETHI airborne SAR and R^2^ = 0.68 for LiDAR data. Table 7 presents examples of studies which utilised fused images for species diversity estimation using both categorical and continuous-scale approaches.

**Table 7:** Examples of studies that classified species types in savanna environment using categorical/continuous-scale approaches.

Savana Biome Ecoregion	Number of species	Area covered	Sensor (Platform)	Categorical/Continuous-scale parameter	Method	Result	Reference
Montane	One species	0.5 km x 2 km	Pléiades-1A, RapidEye and Landsat-8 (Spaceborne)	Tree detection	Object based classification	Overall accuracy (63%–90%)	[201]
Temperate	One species and one land cover type	52 km^2^	Sentinel-1 C-band and LiDAR Waveform (Spaceborne and Airborne)	Class separability	RF	Overall accuracy (30%–60%)	[202]
Flooded	18 species	869 ha	Trimble Harrier 68i laser scanner data and LiDAR (Spaceborne)	Individual tree extraction	CNN	Overall accuracy = 73%	[203]
Tropical	Five species	580 km^2^	RapidEye, Landsat-5 and TerraSAR-X (Spaceborne)	Class separability	RF	Overall Accuracy (89%–94%)	[204]
Temperate	110 species	9 km^2^	RapidEye and LiDAR Small-foot-print 1.5 points/m^2^ (Spaceborne and Airborne)	GLCM, EVI, NDVI and Individual tree extraction	All-possible subset regression	R^2^ (0.68–0.89)	[35]
Montane	Three species and two land cover types	35 000 ha	RADARSAT-2 and waveform LiDAR (Spaceborne and Airborne)	Individual tree extraction	Regression analysis	R^2^ (0.67–0.75)	[205]
Montane	Four species	239 km^2^	Hyperspectral (HyMap, HyVista, Inc.) and Waveform LiDAR (Spaceborne)	25 spectral variables and Individual tree extraction	Regression analysis	R^2^ (0.63–0.71)	[206]
Tropical	Three species	37 ha	UAS, Sentinel-1 C-band and Sentinel-2A (Spaceborne)	10 spectral variables	RF regression model	R^2^ (0.70–0.90)	[207]
Montane	Six species and four land cover types	101 800 km^2^	ALOS PALSAR Landsat-7 (Spaceborne)	HH and HV Mean spectral reflectance	Stepwise regression analysis	R^2^ (0.28 to 0.44)	[179]

## 7. Conclusions and possible future potentials of remotely sensed data

The literature review analysed previous works which utilised optical, RADAR and LiDAR remote sensing for the assessment of woody plant species diversity in different savanna environments. A number of studies have used categorical classification methods to identify a discrete number of species at mixed accuracy levels, depending chiefly on remotely-sensed data characteristics and the species of interest. Alternative assessment methods that convert categorical species data into continuous diversity scale eliminate the restriction on the number of species that can be estimated. Such methods have been applied widely in the assessment of savanna woody plant species. Both methods utilised optical (multispectral and hyperspectral) and structural (LiDAR and RADAR) data. In this regard, although single sensor datasets are the most common image source, fusing different datasets is becoming an attractive option in savanna woody species classification. Data fusion is preferred because it exploits the benefits of more than one dataset, and the fact that fusions that involve RADAR data enable all-year round assessment [190].

Given the evidence in the literature, we recommend studies in the following areas of interest:

- Methods for routine application of structural and optical remote sensing should be expanded to assess diversity amongst physiologically similar woody plant species.

- Studies on discriminating woody plant species at the relatively fine spatial resolution afforded by UASs should be promoted. This will enable identification of complexities due to seasonal dynamics and landscape heterogeneity.

- Image fusion should be explored as a means of improving the accuracy of assessing woody plant species diversity at both improved spectral and spatial resolutions, particularly in areas such as African savannas that are affected by seasonal weather changes.
- Limitations in spectral resolutions in most publicly available remotely-sensed data such as the Landsat and Sentinel multispectral series is acknowledged in the effort to accurately classify plants in species-diverse environments. There is therefore the need to expand the provision and exploration of hyperspectral images at affordable or no costs.
- Woody plant species of the same vegetation environment can have different ages, growing conditions, sizes and shapes that can lead to considerable within-species variability in woody plant species spectral characteristics. There is therefore the need for LiDAR, RADAR and optical images with high temporal resolution to effectively assess species diversity in savanna.
- Multi-temporal analysis of remotely sensed data should be widely explored as a means of improving species diversity in savanna environments that exhibits subtle differences in physiologically similar plant species.
- Since woody plant species phenology varies, species-specific knowledge of phenology is imperative and desirable to inform choice of remotely sensed data with acquisition dates in line with phenological cycle of species under investigation.

## Supporting information

S1 Checklist PRISMA 2009 checklist. (DOCX)

S1 Table Quality assessment scores based on AMSTAR2 for review of reviews. (PDF)

## References

[1] Wakeling JL, Cramer MD, Bond WJ. The savanna-grassland “treeline”: Why don’t savanna trees occur in upland grasslands? J Ecol. 2012;100. doi: 10.1111/j.1365-2745.2011.01921.x

[2] Mucina L, Rutherford, Michael C. Powrie LW. Biomes and Bioregions of Southern Africa. Veg South Africa, Lesotho Swaziland Strelitzia 19. 2006. doi: 10.1289/ehp.7863

[3] Pennington RT, Lehmann CER, Rowland LM. Tropical savannas and dry forests. Curr Bio. 2018. doi: 10.1016/j.cub.2018.03.014 29738723

[4] Tietjen B. Same rainfall amount different vegetation-How environmental conditions and their interactions influence savanna dynamics. Ecol Modell. 2016. doi: 10.1016/j.ecolmodel.2015.06.013

[5] D’Onofrio D, Sweeney L, von Hardenberg J, Baudena M. Grass and tree cover responses to intra-seasonal rainfall variability vary along a rainfall gradient in African tropical grassy biomes. Sci Rep. 2019. doi: 10.1038/s41598-019-38933-9 30787370 PMC6382848

[6] Olson DM, Dinerstein E, Wikramanayake ED, Burgess ND, Powell GVN, Underwood EC, et al. Terrestrial ecoregions of the world: A new map of life on Earth. BioScience. 2001. doi: 10.1641/0006-3568(2001)051[0933:TEOTWA]2.0.CO;2

[7] Archibald S, Bond WJ, Hoffmann W, Lehmann C, Staver C, Stevens N. Distribution and Determinants of Savannas. Savanna Woody Plants and Large Herbivores. 2019. doi: 10.1002/9781119081111.ch1

[8] Weiner T, Gross A, Moreno G, Migliavacca M, Schrumpf M, Reichstein M, et al. Following the Turnover of Soil Bioavailable Phosphate in Mediterranean Savanna by Oxygen Stable Isotopes. J Geophys Res Biogeosciences. 2018. doi: 10.1029/2017JG004086

[9] Sharp BR, Whittaker RJ. The irreversible cattle-driven transformation of a seasonally flooded Australian savanna. J Biogeogr. 2003. doi: 10.1046/j.1365-2699.2003.00840.x

[10] Bush MB, Hanselman JA, Hooghiemstra H. Andean montane forests and climate change. Tropical Rainforest Responses to Climatic Change. 2011. doi: 10.1007/978-3-642-05383-2_2

[11] Archer SR, Andersen EM, Predick KI, Schwinning S, Steidl RJ, Woods SR. Woody Plant Encroachment: Causes and Consequences. 2017. doi: 10.1007/978-3-319-46709-2_2

[12] Sankaran M. Droughts and the ecological future of tropical savanna vegetation. Journal of Ecology. 2019. doi: 10.1111/1365-2745.13195

[13] Smith WK, Dannenberg MP, Yan D, Herrmann S, Barnes ML, Barron-Gafford GA, et al. Remote sensing of dryland ecosystem structure and function: Progress, challenges, and opportunities. Remote Sens Environ. 2019. doi: 10.1016/j.rse.2019.111401

[14] Sankaran M, Ratnam J, Hanan NP. Tree-grass coexistence in savannas revisited—Insights from an examination of assumptions and mechanisms invoked in existing models. Ecology Letters. 2004. doi: 10.1111/j.1461-0248.2004.00596.x

[15] Hill MJ, Hill MJ. Savanna Biome. Ecology. 2013. doi: 10.1093/obo/9780199830060-0043

[16] Landmann T, Piiroinen R, Makori DM, Abdel-Rahman EM, Makau S, Pellikka P, et al. Application of hyperspectral remote sensing for flower mapping in African savannas. Remote Sens Environ. 2015;166: 50–60. doi: 10.1016/j.rse.2015.06.006

[17] Ondier J, Okach DO, John OC, Otieno DO. Influence of rainfall amount and livestock grazing on soil respiration in a moist Kenyan savannah. Afr J Ecol. 2020. doi: 10.1111/aje.12670

[18] Anadón JD, Sala OE, Maestre FT. Climate change will increase savannas at the expense of forests and treeless vegetation in tropical and subtropical Americas. J Ecol. 2014;102. doi: 10.1111/1365-2745.12325

[19] Ma X, Huete A, Yu Q, Coupe NR, Davies K, Broich M, et al. Spatial patterns and temporal dynamics in savanna vegetation phenology across the north australian tropical transect. Remote Sens Environ. 2013;139. doi: 10.1016/j.rse.2013.07.030

[20] Moore N, Alagarswamy G, Pijanowski B, Thornton P, Lofgren B, Olson J, et al. East African food security as influenced by future climate change and land use change at local to regional scales. Clim Change. 2012;110: 823–844. doi: 10.1007/S10584-011-0116-7

[21] Tews J, Jeltsch F. Modelling the impact of climate change on woody plant population dynamics in South African savanna. BMC Ecol. 2004. doi: 10.1186/1472-6785-4-17 15606921 PMC544358

[22] Marchant R. Understanding complexity in savannas: Climate, biodiversity and people. Current Opinion in Environmental Sustainability. 2010. doi: 10.1016/j.cosust.2010.03.001

[23] Chidumayo EN. Implications of climate warming on seedling emergence and mortality of African savanna woody plants. Plant Ecol. 2008. doi: 10.1007/s11258-007-9385-7

[24] Karlson M, Ostwald M. Remote sensing of vegetation in the Sudano-Sahelian zone: A literature review from 1975 to 2014. Journal of Arid Environments. 2016. doi: 10.1016/j.jaridenv.2015.08.022

[25] Zhang W, Brandt M, Wang Q, Prishchepov A V., Tucker CJ, Li Y, et al. From woody cover to woody canopies: How Sentinel-1 and Sentinel-2 data advance the mapping of woody plants in savannas. Remote Sens Environ. 2019;234: 111465. doi: 10.1016/j.rse.2019.111465

[26] Naidoo L, Mathieu R, Main R, Kleynhans W, Wessels K, Asner G, et al. Savannah woody structure modelling and mapping using multi-frequency (X-, C- and L-band) Synthetic Aperture Radar data. ISPRS J Photogramm Remote Sens. 2015. doi: 10.1016/j.isprsjprs.2015.04.007

[27] Madonsela S, Cho MA, Ramoelo A, Mutanga O, Naidoo L. Estimating tree species diversity in the savannah using NDVI and woody canopy cover. Int J Appl Earth Obs Geoinf. 2018;66: 106–115. doi: 10.1016/j.jag.2017.11.005

[28] Moore CE, Brown T, Keenan TF, Duursma RA, Van Dijk AIJM, Beringer J, et al. Reviews and syntheses: Australian vegetation phenology: New insights from satellite remote sensing and digital repeat photography. Biogeosciences. 2016. doi: 10.5194/bg-13-5085-2016

[29] Pohl C, Van Genderen JL. Review article Multisensor image fusion in remote sensing: Concepts, methods and applications. International Journal of Remote Sensing. 1998. doi: 10.1080/014311698215748

[30] Maxwell AE, Warner TA, Fang F. Implementation of machine-learning classification in remote sensing: An applied review. International Journal of Remote Sensing. 2018. doi: 10.1080/01431161.2018.1433343

[31] Gaughan AE, Stevens FR, Gibbes C, Southworth J, Binford MW. Linking vegetation response to seasonal precipitation in the Okavango-Kwando-Zambezi catchment of southern Africa. Int J Remote Sens. 2012. doi: 10.1080/01431161.2012.692831

[32] Evans TL, Costa M. Landcover classification of the Lower Nhecolândia subregion of the Brazilian Pantanal Wetlands using ALOS/PALSAR, RADARSAT-2 and ENVISAT/ASAR imagery. Remote Sens Environ. 2013. doi: 10.1016/j.rse.2012.09.022

[33] Möckel T, Dalmayne J, Schmid BC, Prentice HC, Hall K. Airborne hyperspectral data predict fine-scale plant species diversity in grazed dry grasslands. Remote Sens. 2016. doi: 10.3390/rs8020133

[34] Furtado LF de A, Silva TSF, Novo EML de M. Dual-season and full-polarimetric C band SAR assessment for vegetation mapping in the Amazon várzea wetlands. Remote Sens Environ. 2016;174: 212–222. doi: 10.1016/j.rse.2015.12.013

[35] George-Chacon SP, Dupuy JM, Peduzzi A, Hernandez-Stefanoni JL. Combining high resolution satellite imagery and lidar data to model woody species diversity of tropical dry forests. Ecol Indic. 2019;101: 975–984. doi: 10.1016/j.ecolind.2019.02.015

[36] Gyamfi-Ampadu E, Gebreslasie M, Mendoza-Ponce A. Mapping natural forest cover using satellite imagery of Nkandla forest reserve, KwaZulu-Natal, South Africa. Remote Sens Appl Soc Environ. 2020. doi: 10.1016/j.rsase.2020.100302

[37] Adole T, Dash J, Atkinson PM. A systematic review of vegetation phenology in Africa. Ecological Informatics. 2016. doi: 10.1016/j.ecoinf.2016.05.004

[38] Ashton P, Zhu H. The tropical-subtropical evergreen forest transition in East Asia: An exploration. Plant Diversity. 2020. doi: 10.1016/j.pld.2020.04.001 33094198 PMC7567766

[39] Ganivet E, Bloomberg M. Towards rapid assessments of tree species diversity and structure in fragmented tropical forests: A review of perspectives offered by remotely-sensed and field-based data. Forest Ecology and Management. 2019. doi: 10.1016/j.foreco.2018.09.003

[40] Gardon FR, Santos RF dos, Rodrigues RR. Brazil’s forest restoration, biomass and carbon stocks: A critical review of the knowledge gaps. Forest Ecology and Management. 2020. doi: 10.1016/j.foreco.2020.117972

[41] Mutanga O, Dube T, Ahmed F. Progress in remote sensing: vegetation monitoring in South Africa. South African Geogr J. 2016. doi: 10.1080/03736245.2016.1208586

[42] Wei M, Qiao B, Zhao J, Zuo X. The area extraction of winter wheat in mixed planting area based on Sentinel-2 a remote sensing satellite images. Int J Parallel, Emergent Distrib Syst. 2020;35: 297–308. doi: 10.1080/17445760.2019.1597084

[43] Santos LCM, Bitencourt MD. Remote sensing in the study of Brazilian mangroves: Review, gaps in the knowledge, new perspectives and contributions for management. Journal of Integrated Coastal Zone Management. 2016. doi: 10.5894/rgci662

[44] De Menezes MPM, Berger U, Mehlig U. Mangrove vegetation in Amazonia: A review of studies from the coast of Pará and Maranhão States, north Brazil. Acta Amaz. 2008;38. doi: 10.1590/S0044-59672008000300004

[45] Sebata A. Ecology of Woody Plants in African Savanna Ecosystems. Plant Ecology—Traditional Approaches to Recent Trends. 2017. doi: 10.5772/intechopen.69865

[46] Liberati A, Altman DG, Tetzlaff J, Mulrow C, Gøtzsche PC, Ioannidis JPA, et al. The PRISMA statement for reporting systematic reviews and meta-analyses of studies that evaluate health care interventions: Explanation and elaboration. PLoS Medicine. 2009. doi: 10.1371/journal.pmed.1000100 19621070 PMC2707010

[47] Gubarev V V., Gorshenkov AA, Klikushin YN, Kobenko VY. Classification measurements: Methods and implementation. Optoelectron Instrum Data Process. 2013;49. doi: 10.3103/s875669901302009x

[48] Fundisi E, Musakwa W, Ahmed FB, Tesfamichael SG. Estimation of woody plant species diversity during a dry season in a savanna environment using the spectral and textural information derived from WorldView-2 imagery. PLoS One. 2020;15. doi: 10.1371/journal.pone.0234158 32511261 PMC7279610

[49] Glatthorn J, Feldmann E, Tabaku V, Leuschner C, Meyer P. Classifying development stages of primeval European beech forests: Is clustering a useful tool? BMC Ecol. 2018;18. doi: 10.1186/s12898-018-0203-y 30458749 PMC6247681

[50] Shannon CE. The Mathematical Theory of Communication. M.D. Computing. 1963. doi: 10.2307/410457 9230594

[51] Colwell RK, Coddington JA. Estimating terrestrial biodiversity through extrapolation. Philos Trans R Soc Lond B Biol Sci. 1994. doi: 10.1098/rstb.1994.0091 7972351

[52] Simpson EH. Measurement of diversity. Nature. 1949. doi: 10.1038/163688a0

[53] Fu W, Ma J, Chen P, Chen F. Remote Sensing Satellites for Digital Earth. Manual of Digital Earth. 2020. doi: 10.1007/978-981-32-9915-3_3

[54] Lillesand TM, Kiefer RW, Chipman JW. Remote sensing and image interpretation. Nev York Chichester Brisbane Toronto 6IS s. John Wiley & Sons; 2004. Available: http://www.osti.gov/energycitations/product.biblio.jsp?osti_id=6028047

[55] Fassnacht FE, Latifi H, Stereńczak K, Modzelewska A, Lefsky M, Waser LT, et al. Review of studies on tree species classification from remotely sensed data. Remote Sens of Env. 2016. doi: 10.1016/j.rse.2016.08.013

[56] Zhang J. Multi-source remote sensing data fusion: Status and trends. International J of Image and Data Fusion. 2010. doi: 10.1080/19479830903561035

[57] Tou Julius and Gonzalez R 197. Pattern recognition principles. Volume 7 of Applied Mathematics and Computation. Addison-Wesley Publishing Company; 1974. p. p377.

[58] Settle JJ, Briggs SA. Fast maximum likelihood classification of remotely-sensed imagery. Int J Remote Sens. 1987;8. doi: 10.1080/01431168708948683

[59] Atkinson PM, Tatnall ARL. Introduction neural networks in remote sensing. Int J Remote Sens. 1997;18. doi: 10.1080/014311697218700

[60] Boggs GS. Assessment of SPOT 5 and QuickBird remotely sensed imagery for mapping tree cover in savannas. Int J Appl Earth Obs Geoinf. 2010. doi: 10.1016/j.jag.2009.11.001

[61] Marston CG, Aplin P, Wilkinson DM, Field R, O’Regan HJ. Scrubbing up: Multi-scale investigation of woody encroachment in a Southern African savannah. Remote Sens. 2017;9. doi: 10.3390/rs9050419

[62] Xie Y, Sha Z, Yu M. Remote sensing imagery in vegetation mapping: a review. J Plant Ecol. 2008. doi: 10.1093/jpe/rtm005

[63] Palamuleni L, Annegarn H, Kneen M, Landmann T. Mapping rural savanna woodlands in Malawi: A comparison of maximum likelihood and fuzzy classifiers. International Geoscience and Remote Sensing Symposium (IGARSS). 2007. doi: 10.1109/IGARSS.2007.4423035

[64] Martins GB, La Rosa LEC, Happ PN, Filho LCTC, Santos CJF, Feitosa RQ, et al. Deep learning-based tree species mapping in a highly diverse tropical urban setting. Urban For Urban Green. 2021;64. doi: 10.1016/j.ufug.2021.127241

[65] Lelong CCD, Tshingomba UK, Soti V. Assessing Worldview-3 multispectral imaging abilities to map the tree diversity in semi-arid parklands. Int J Appl Earth Obs Geoinf. 2020. doi: 10.1016/j.jag.2020.102211

[66] Breiman L. Random forests. Mach Learn. 2001. doi: 10.1023/A:1010933404324

[67] Cortes C, Vapnik V. Support-Vector Networks. Mach Learn. 1995. doi: 10.1023/A:1022627411411

[68] Quinlan JR. Induction of decision trees. Mach Learn. 1986;1: 81–106.

[69] Freund Y, Schapire RE. Experiments with a New Boosting Algorithm. Proc 13th Int Conf Mach Learn. 1996. doi: 10.1.1.133.1040

[70] Schmidhuber J. Deep Learning in neural networks: An overview. Neural Networks. 2015. doi: 10.1016/j.neunet.2014.09.003 25462637

[71] Krizhevsky A, Sutskever I, Hinton GE. ImageNet classification with deep convolutional neural networks. Commun ACM. 2017;60. doi: 10.1145/3065386

[72] Kganyago M, Odindi J, Adjorlolo C, Mhangara P. Evaluating the capability of Landsat 8 OLI and SPOT 6 for discriminating invasive alien species in the African Savanna landscape. Int J Appl Earth Obs Geoinf. 2018;67: 10–19. doi: 10.1016/j.jag.2017.12.008

[73] Wendelberger KS, Gann D, Richards JH. Using Bi-seasonal worldview-2 multi-spectral data and supervised random forest classification to map coastal plant communities in everglades national park. Sensors (Switzerland). 2018. doi: 10.3390/s18030829 29522476 PMC5876715

[74] Zhang M, Li W, Du Q. Diverse region-based CNN for hyperspectral image classification. IEEE Trans Image Process. 2018;27. doi: 10.1109/TIP.2018.2809606 29533899

[75] dos Santos AA, Marcato Junior J, Araújo MS, Di Martini DR, Tetila EC, Siqueira HL, et al. Assessment of CNN-based methods for individual tree detection on images captured by RGB cameras attached to UAVS. Sensors (Switzerland). 2019;19. doi: 10.3390/s19163595 31426597 PMC6719170

[76] Cho MA, Malahlela O, Ramoelo A. Assessing the utility WorldView-2 imagery for tree species mapping in South African subtropical humid forest and the conservation implications: Dukuduku forest patch as case study. Int J Appl Earth Obs Geoinf. 2015. doi: 10.1016/j.jag.2015.01.015

[77] Feret JB, Asner GP. Tree species discrimination in tropical forests using airborne imaging spectroscopy. IEEE Trans Geosci Remote Sens. 2013;51: 73–84. doi: 10.1109/TGRS.2012.2199323

[78] Lin C, Popescu SC, Thomson G, Tsogt K, Chang CI. Classification of tree species in overstorey canopy of subtropical forest using QuickBird images. PLoS One. 2015;10: 1–23. doi: 10.1371/journal.pone.0125554 25978466 PMC4433356

[79] Ouma YO, Tetuko J, Tateishi R. Analysis of co-occurrence and discrete wavelet transform textures for differentiation of forest and non-forest vegetation in very-high-resolution optical-sensor imagery. Int J Remote Sens. 2008. doi: 10.1080/01431160701601782

[80] Madonsela S, Cho MA, Mathieu R, Mutanga O, Ramoelo A, Kaszta Ż, et al. Multi-phenology WorldView-2 imagery improves remote sensing of savannah tree species. Int J Appl Earth Obs Geoinf. 2017;58: 65–73. doi: 10.1016/j.jag.2017.01.018

[81] Ferreira MP, Zortea M, Zanotta DC, Shimabukuro YE, de Souza Filho CR. Mapping tree species in tropical seasonal semi-deciduous forests with hyperspectral and multispectral data. Remote Sens Environ. 2016;179: 66–78. doi: 10.1016/j.rse.2016.03.021

[82] Macintyre P, van Niekerk A, Mucina L. Efficacy of multi-season Sentinel-2 imagery for compositional vegetation classification. Int J Appl Earth Obs Geoinf. 2020. doi: 10.1016/j.jag.2019.101980

[83] Forkuor G, Dimobe K, Serme I, Tondoh JE. Landsat-8 vs. Sentinel-2: examining the added value of sentinel-2’s red-edge bands to land-use and land-cover mapping in Burkina Faso. GIScience Remote Sens. 2018;55: 331–354. doi: 10.1080/15481603.2017.1370169

[84] Lewis D, Phinn S, Pfitzner K. Pixel-based image classification to map vegetation communities using SPOT5 and Landsat5 thematic mapper data in a tropical savanna, northern Australia. Can J Remote Sens. 2012;38: 570–585. doi: 10.5589/m12-047

[85] chien Shih H, Stow DA, Tsai YH. Guidance on and comparison of machine learning classifiers for Landsat-based land cover and land use mapping. Int J Remote Sens. 2019;40: 1248–1274. doi: 10.1080/01431161.2018.1524179

[86] Vreugdenhil M, Wagner W, Bauer-Marschallinger B, Pfeil I, Teubner I, Rüdiger C, et al. Sensitivity of Sentinel-1 backscatter to vegetation dynamics: An Austrian case study. Remote Sens. 2018. doi: 10.3390/rs10091396

[87] Shen X, Wang D, Mao K, Anagnostou E, Hong Y. Inundation extent mapping by synthetic aperture radar: A review. Remote Sens. 2019;11: 1–18. doi: 10.3390/RS11070879

[88] Zhu L, Suomalainen J, Liu J, Hyyppä J, Kaartinen H, Haggren H. A Review: Remote Sensing Sensors. Multi-purposeful Application of Geospatial Data. 2018. doi: 10.5772/intechopen.71049

[89] Kulkarni SC, Rege PP. Pixel level fusion techniques for SAR and optical images: A review. Inf Fusion. 2020;59: 13–29. doi: 10.1016/j.inffus.2020.01.003

[90] Nagraj GM, Karegowda AG. Crop Mapping using SAR Imagery: An Review. Int J Adv Res Comput Sci. 2016.

[91] Morandeira NS, Grings F, Facchinetti C, Kandus P. Mapping plant functional types in floodplain wetlands: An analysis of C-band polarimetric SAR data from RADARSAT-2. Remote Sens. 2016;8. doi: 10.3390/rs8030174

[92] Dickinson C, Siqueira P, Clewley D, Lucas R. Classification of forest composition using polarimetric decomposition in multiple landscapes. Remote Sens Environ. 2013;131. doi: 10.1016/j.rse.2012.12.013

[93] Dong X, Quegan S, Yumiko U, Hu C, Zeng T. Feasibility study of C- and L-band SAR time series data in tracking Indonesian plantation and natural forest cover changes. IEEE J Sel Top Appl Earth Obs Remote Sens. 2015;8. doi: 10.1109/JSTARS.2015.2400439

[94] Kuria DN, Menz G, Misana S, Mwita E, Thamm H-P, Alvarez M, et al. Seasonal Vegetation Changes in the Malinda Wetland Using Bi-Temporal, Multi-Sensor, Very High Resolution Remote Sensing Data Sets. Adv Remote Sens. 2014;03. doi: 10.4236/ars.2014.31004

[95] Nicolau AP, Flores-Anderson A, Griffin R, Herndon K, Meyer FJ. Assessing SAR C-band data to effectively distinguish modified land uses in a heavily disturbed Amazon forest. Int J Appl Earth Obs Geoinf. 2021. doi: 10.1016/j.jag.2020.102214

[96] Jin H, Mountrakis G, Stehman S V. Assessing integration of intensity, polarimetric scattering, interferometric coherence and spatial texture metrics in PALSAR-derived land cover classification. ISPRS J Photogramm Remote Sens. 2014;98. doi: 10.1016/j.isprsjprs.2014.09.017

[97] Prudente VHR, Sanches ID, Adami M, Skakun S, Oldoni L V., Xaud HAM, et al. SAR Data for Land Use Land Cover Classification in a Tropical Region with Frequent Cloud Cover. International Geoscience and Remote Sensing Symposium (IGARSS). 2020. doi: 10.1109/IGARSS39084.2020.9323404

[98] Ho Tong Minh D, Ienco D, Gaetano R, Lalande N, Ndikumana E, Osman F, et al. Deep Recurrent Neural Networks for Winter Vegetation Quality Mapping via Multitemporal SAR Sentinel-1. IEEE Geosci Remote Sens Lett. 2018;15: 465–468. doi: 10.1109/LGRS.2018.2794581

[99] Ge S, Antropov O, Su W, Gu H, Praks J. Deep recurrent neural networks for land-cover classification using sentinel-1 insar time series. International Geoscience and Remote Sensing Symposium (IGARSS). 2019. doi: 10.1109/IGARSS.2019.8900088

[100] Niu X, Ban Y. Multi-temporal RADARSAT-2 polarimetric SAR data for urban land-cover classification using an object-based support vector machine and a rule-based approach. Int J Remote Sens. 2013. doi: 10.1080/01431161.2012.700133

[101] Tsyganskaya V, Martinis S, Marzahn P, Ludwig R. Detection of temporary flooded vegetation using Sentinel-1 time series data. Remote Sens. 2018. doi: 10.3390/rs10081286

[102] Canisius F, Brisco B, Murnaghan K, Van Der Kooij M, Keizer E. SAR backscatter and InSAR coherence for monitoring wetland extent, flood pulse and vegetation: A study of the Amazon lowland. Remote Sens. 2019. doi: 10.3390/RS11060720

[103] Qi Z, Yeh AGO, Li X, Lin Z. A novel algorithm for land use and land cover classification using RADARSAT-2 polarimetric SAR data. Remote Sens Environ. 2012. doi: 10.1016/j.rse.2011.11.001

[104] Camargo FF, Sano EE, Almeida CM, Mura JC, Almeida T. A comparative assessment of machine-learning techniques for land use and land cover classification of the Brazilian tropical savanna using ALOS-2/PALSAR-2 polarimetric images. Remote Sens. 2019. doi: 10.3390/rs11131600

[105] Ferreira-Ferreira J, Silva TSF, Streher AS, Affonso AG, De Almeida Furtado LF, Forsberg BR, et al. Combining ALOS/PALSAR derived vegetation structure and inundation patterns to characterize major vegetation types in the Mamirauá Sustainable Development Reserve, Central Amazon floodplain, Brazil. Wetl Ecol Manag. 2014. doi: 10.1007/s11273-014-9359-1

[106] Liesenberg V, Gloaguen R. Evaluating SAR polarization modes at L-band for forest classification purposes in eastern Amazon, Brazil. Int J Appl Earth Obs Geoinf. 2012. doi: 10.1016/j.jag.2012.08.016

[107] Haarpaintner J, Einzmann K, Pedrazzani D, Mateos San Juan MT, Gómez Giménez M, Heinzel J, et al. Tropical forest remote sensing services for the Democratic Republic of Congo case inside the EU FP7 “ReCover” project (1st iteration). International Geoscience and Remote Sensing Symposium (IGARSS). 2012. doi: 10.1109/IGARSS.2012.6352722

[108] Lim K, Treitz P, Wulder M, St-Ongé B, Flood M. LiDAR remote sensing of forest structure. Prog Phys Geogr. 2003. doi: 10.1191/0309133303pp360ra

[109] Hyyppä J, Hyyppä H, Leckie D, Gougeon F, Yu X, Maltamo M. Review of methods of small-footprint airborne laser scanning for extracting forest inventory data in boreal forests. International Journal of Remote Sensing. 2008. doi: 10.1080/01431160701736489

[110] Wulder MA, White JC, Nelson RF, Næsset E, Ørka HO, Coops NC, et al. Lidar sampling for large-area forest characterization: A review. Remote Sensing of Environment. 2012. doi: 10.1016/j.rse.2012.02.001

[111] Fedrigo M, Newnham GJ, Coops NC, Culvenor DS, Bolton DK, Nitschke CR. Predicting temperate forest stand types using only structural profiles from discrete return airborne lidar. ISPRS J Photogramm Remote Sens. 2018. doi: 10.1016/j.isprsjprs.2017.11.018

[112] Dowling R, Accad A. Vegetation classification of the riparian zone along the Brisbane River, Queensland, Australia, using light detection and ranging (lidar) data and forward looking digital video. Can J Remote Sens. 2003;29. doi: 10.5589/m03-029

[113] Morsdorf F, Mårell A, Koetz B, Cassagne N, Pimont F, Rigolot E, et al. Discrimination of vegetation strata in a multi-layered Mediterranean forest ecosystem using height and intensity information derived from airborne laser scanning. Remote Sens Environ. 2010;114. doi: 10.1016/j.rse.2010.01.023

[114] Falkowski MJ, Evans JS, Martinuzzi S, Gessler PE, Hudak AT. Characterizing forest succession with lidar data: An evaluation for the Inland Northwest, USA. Remote Sens Environ. 2009. doi: 10.1016/j.rse.2009.01.003

[115] Li Z, Strahler A, Schaaf C, Jupp D, Schaefer M, Olofsson P. Seasonal change of leaf and woody area profiles in a midlatitude deciduous forest canopy from classified dual-wavelength terrestrial lidar point clouds. Agric For Meteorol. 2018. doi: 10.1016/j.agrformet.2018.07.014

[116] Lopatin J, Dolos K, Hernández HJ, Galleguillos M, Fassnacht FE. Comparing Generalized Linear Models and random forest to model vascular plant species richness using LiDAR data in a natural forest in central Chile. Remote Sens Environ. 2016;173. doi: 10.1016/j.rse.2015.07.026

[117] Guan H, Yu Y, Ji Z, Li J, Zhang Q. Deep learning-based tree classification using mobile LiDAR data. Remote Sens Lett. 2015. doi: 10.1080/2150704X.2015.1088668

[118] Hamraz H, Jacobs NB, Contreras MA, Clark CH. Deep learning for conifer/deciduous classification of airborne LiDAR 3D point clouds representing individual trees. ISPRS J Photogramm Remote Sens. 2019. doi: 10.1016/j.isprsjprs.2019.10.011

[119] Brandtberg T. Classifying individual tree species under leaf-off and leaf-on conditions using airborne lidar. ISPRS J Photogramm Remote Sens. 2007. doi: 10.1016/j.isprsjprs.2006.10.006

[120] Liu L, Coops NC, Aven NW, Pang Y. Mapping urban tree species using integrated airborne hyperspectral and LiDAR remote sensing data. Remote Sens Environ. 2017. doi: 10.1016/j.rse.2017.08.010

[121] Marselis SM, Tang H, Armston JD, Calders K, Labrière N, Dubayah R. Distinguishing vegetation types with airborne waveform lidar data in a tropical forest-savanna mosaic: A case study in Lopé National Park, Gabon. Remote Sens Environ. 2018. doi: 10.1016/j.rse.2018.07.023

[122] Ilangakoon NT, Glenn NF, Dashti H, Painter TH, Mikesell TD, Spaete LP, et al. Constraining plant functional types in a semi-arid ecosystem with waveform lidar. Remote Sens Environ. 2018. doi: 10.1016/j.rse.2018.02.070

[123] Cao L, Coops NC, Hermosilla T, Innes J, Dai J, She G. Using small-footprint discrete and full-waveform airborne LiDAR metrics to estimate total biomass and biomass components in subtropical forests. Remote Sens. 2014. doi: 10.3390/rs6087110

[124] Alexander C, Deák B, Kania A, Mücke W, Heilmeier H. Classification of vegetation in an open landscape using full-waveform airborne laser scanner data. Int J Appl Earth Obs Geoinf. 2015. doi: 10.1016/j.jag.2015.04.014

[125] Fieber KD, Davenport IJ, Ferryman JM, Gurney RJ, Walker JP, Hacker JM. Analysis of full-waveform LiDAR data for classification of an orange orchard scene. ISPRS J Photogramm Remote Sens. 2013. doi: 10.1016/j.isprsjprs.2013.05.002

[126] Arekhi M, Yılmaz OY, Yılmaz H, Akyüz YF. Can tree species diversity be assessed with Landsat data in a temperate forest? Environ Monit Assess. 2017. doi: 10.1007/s10661-017-6295-6 29080961

[127] Mapfumo RB, Murwira A, Masocha M, Andriani R. Detection of subtle deforestation due to logging using satellite remote sensing in wet and dry savanna woodlands of Southern Africa. Geocarto Int. 2017;32: 514–530. doi: 10.1080/10106049.2016.1161074

[128] Rocchini D, Balkenhol N, Carter GA, Foody GM, Gillespie TW, He KS, et al. Remotely sensed spectral heterogeneity as a proxy of species diversity: Recent advances and open challenges. Ecol Inform. 2010. doi: 10.1016/j.ecoinf.2010.06.001

[129] Hall-Beyer M. Practical guidelines for choosing GLCM textures to use in landscape classification tasks over a range of moderate spatial scales. Int J Remote Sens. 2017. doi: 10.1080/01431161.2016.1278314

[130] Wood EM, Pidgeon AM, Radeloff VC, Keuler NS. Image texture as a remotely sensed measure of vegetation structure. Remote Sens Environ. 2012;121: 516–526. doi: 10.1016/j.rse.2012.01.003

[131] Zhou J, Yan Guo R, Sun M, Di TT, Wang S, Zhai J, et al. The Effects of GLCM parameters on LAI estimation using texture values from Quickbird Satellite Imagery. Sci Rep. 2017;7. doi: 10.1038/s41598-017-07951-w 28779107 PMC5544764

[132] Madonsela S, Cho MA, Ramoelo A, Mutanga O. Remote sensing of species diversity using Landsat 8 spectral variables. ISPRS J Photogramm Remote Sens. 2017. doi: 10.1016/j.isprsjprs.2017.10.008

[133] Gallardo-Cruz JA, Meave JA, González EJ, Lebrija-Trejos EE, Romero-Romero MA, Pérez-García EA, et al. Predicting tropical dry forest successional attributes from space: Is the key hidden in image texture? PLoS One. 2012;7. doi: 10.1371/journal.pone.0030506 22363443 PMC3282724

[134] Warner TA, Skowronski NS, Gallagher MR. High spatial resolution burn severity mapping of the New Jersey Pine Barrens with WorldView-3 near-infrared and shortwave infrared imagery. Int J Remote Sens. 2017. doi: 10.1080/01431161.2016.1268739

[135] Zhou J, Dian Y, Wang X, Yao C, Jian Y, Li Y, et al. Comparison of GF2 and SPOT6 imagery on canopy cover estimating in northern subtropics forest in China. Forests. 2020;11. doi: 10.3390/F11040407

[136] Nguyen TD, Kappas M. Estimating the aboveground biomass of an evergreen broadleaf forest in Xuan Lien Nature Reserve, Thanh Hoa, Vietnam, using SPOT-6 data and the random forest algorithm. Int J For Res. 2020. doi: 10.1155/2020/4216160

[137] Krofcheck DJ, Eitel JUH, Lippitt CD, Vierling LA, Schulthess U, Litvak ME. Remote sensing based simple models of GPP in both disturbed and undisturbed piñon-juniper woodlands in the southwestern U.S. Remote Sens. 2016. doi: 10.3390/rs8010020

[138] Torresani M, Rocchini D, Sonnenschein R, Zebisch M, Marcantonio M, Ricotta C, et al. Estimating tree species diversity from space in an alpine conifer forest: The Rao’s Q diversity index meets the spectral variation hypothesis. Ecol Inform. 2019. doi: 10.1016/j.ecoinf.2019.04.001

[139] Machado CCC, Galvíncio JD, de Moura MSB, de Araujo HFP. Predicting plant species richness with satellite images in the largest dry forest nucleus in South America. J Arid Environ. 2019;166: 43–50. doi: 10.1016/j.jaridenv.2019.03.001

[140] Tsalyuk M, Kelly M, Getz WM. Improving the prediction of African savanna vegetation variables using time series of MODIS products. ISPRS J Photogramm Remote Sens. 2017. doi: 10.1016/j.isprsjprs.2017.07.012 30739997 PMC6368348

[141] Chen W, Zheng Q, Xiang H, Chen X, Sakai T. Forest canopy height estimation using polarimetric interferometric synthetic aperture radar (Polinsar) technology based on full-polarized alos/palsar data. Remote Sens. 2021;13. doi: 10.3390/rs13020174

[142] Kumar A, Kishore BSPC, Saikia P, Deka J, Bharali S, Singha LB, et al. Tree diversity assessment and above ground forests biomass estimation using SAR remote sensing: A case study of higher altitude vegetation of North-East Himalayas, India. Phys Chem Earth. 2019. doi: 10.1016/j.pce.2019.03.007

[143] Periasamy S. Significance of dual polarimetric synthetic aperture radar in biomass retrieval: An attempt on Sentinel-1. Remote Sens Environ. 2018;217: 537–549. doi: 10.1016/j.rse.2018.09.003

[144] Urbazaev M, Thiel C, Mathieu R, Naidoo L, Levick SR, Smit IPJ, et al. Assessment of the mapping of fractional woody cover in southern African savannas using multi-temporal and polarimetric ALOS PALSAR L-band images. Remote Sens Environ. 2015. doi: 10.1016/j.rse.2015.06.013

[145] Menges CH, Hill GJE, Ahmad W. Use of airborne video data for the characterization of tropical savannas in northern Australia: The optimal spatial resolution for remote sensing applications. Int J Remote Sens. 2001;22. doi: 10.1080/01431160051060129

[146] Kuplich TM, Curran PJ, Atkinson PM. Relating SAR image texture to the biomass of regenerating tropical forests. Int J Remote Sens. 2005;26. doi: 10.1080/01431160500239107

[147] Sarker MLR, Nichol J, Iz HB, Ahmad B Bin, Rahman AA. Forest biomass estimation using texture measurements of high-resolution dual-polarization C-band SAR data. IEEE Trans Geosci Remote Sens. 2013;51. doi: 10.1109/TGRS.2012.2219872

[148] Du C, Liao S, Boufford DE, Ma J. Twenty years of Chinese vascular plant novelties, 2000 through 2019. Plant Divers. 2020;42. doi: 10.1016/j.pld.2020.08.004 33134624 PMC7584791

[149] Kumar S, Khati UG, Chandola S, Agrawal S, Kushwaha SPS. Polarimetric SAR Interferometry based modeling for tree height and aboveground biomass retrieval in a tropical deciduous forest. Adv Sp Res. 2017;60. doi: 10.1016/j.asr.2017.04.018

[150] Van Pham M, Pham TM, Viet Du QV, Bui QT, Van Tran A, Pham HM, et al. Integrating Sentinel-1A SAR data and GIS to estimate aboveground biomass and carbon accumulation for tropical forest types in Thuan Chau district, Vietnam. Remote Sens Appl Soc Environ. 2019. doi: 10.1016/j.rsase.2019.03.003

[151] Bispo PDC, Pardini M, Papathanassiou KP, Kugler F, Balzter H, Rains D, et al. Mapping forest successional stages in the Brazilian Amazon using forest heights derived from TanDEM-X SAR interferometry. Remote Sens Environ. 2019. doi: 10.1016/j.rse.2019.05.013

[152] Huang X, Ziniti B, Torbick N, Ducey MJ. Assessment of forest above ground biomass estimation using multi-temporal C-band Sentinel-1 and Polarimetric L-band PALSAR-2 data. Remote Sens. 2018. doi: 10.3390/rs10091424

[153] Mitchard ETA, Saatchi SS, Lewis SL, Feldpausch TR, Woodhouse IH, Sonké B, et al. Measuring biomass changes due to woody encroachment and deforestation/degradation in a forest-savanna boundary region of central Africa using multi-temporal L-band radar backscatter. Remote Sens Environ. 2011. doi: 10.1016/j.rse.2010.02.022

[154] Ningthoujam RK, Joshi PK, Roy PS. Retrieval of forest biomass for tropical deciduous mixed forest using ALOS PALSAR mosaic imagery and field plot data. Int J Appl Earth Obs Geoinf. 2018. doi: 10.1016/j.jag.2018.03.007

[155] Michelakis D, Stuart N, Brolly M, Woodhouse IH, Lopez G, Linares V. Estimation of woody biomass of pine savanna woodlands from ALOS PALSAR imagery. IEEE J Sel Top Appl Earth Obs Remote Sens. 2015. doi: 10.1109/JSTARS.2014.2365253

[156] Odipo VO, Nickless A, Berger C, Baade J, Urbazaev M, Walther C, et al. Assessment of aboveground woody biomass dynamics using terrestrial laser scanner and L-band ALOS PALSAR data in South African Savanna. Forests. 2016. doi: 10.3390/f7120294

[157] Avtar R, Suzuki R, Takeuchi W, Sawada H. PALSAR 50 m Mosaic Data Based National Level Biomass Estimation in Cambodia for Implementation of REDD+ Mechanism. PLoS One. 2013. doi: 10.1371/journal.pone.0074807 24116012 PMC3792093

[158] Thumaty KC, Fararoda R, Middinti S, Gopalakrishnan R, Jha CS, Dadhwal VK. Estimation of above ground biomass for central Indian deciduous forests using ALOS PALSAR L-band data. J Indian Soc Remote Sens. 2016. doi: 10.1007/s12524-015-0462-4

[159] Hernández-Stefanoni JL, Dupuy JM, Johnson KD, Birdsey R, Tun-Dzul F, Peduzzi A, et al. Improving species diversity and biomass estimates of tropical dry forests using airborne LiDAR. Remote Sens. 2014;6. doi: 10.3390/rs6064741

[160] Simonson WD, Allen HD, Coomes DA. Use of an Airborne Lidar System to Model Plant Species Composition and Diversity of Mediterranean Oak Forests. Conserv Biol. 2012;26. doi: 10.1111/j.1523-1739.2012.01869.x 22731687

[161] Listopad CMCS, Masters RE, Drake J, Weishampel J, Branquinho C. Structural diversity indices based on airborne LiDAR as ecological indicators for managing highly dynamic landscapes. Ecol Indic. 2015;57. doi: 10.1016/j.ecolind.2015.04.017

[162] Tamburlin D, Torresani M, Tomelleri E, Tonon G, Rocchini D. Testing the height variation hypothesis with the R rasterdiv package for tree species diversity estimation. Remote Sens. 2021;13. doi: 10.3390/rs13183569

[163] Teobaldelli M, Cona F, Saulino L, Migliozzi A, D’Urso G, Langella G, et al. Detection of diversity and stand parameters in Mediterranean forests using leaf-off discrete return LiDAR data. Remote Sens Environ. 2017;192. doi: 10.1016/j.rse.2017.02.008

[164] Marselis SM, Abernethy K, Alonso A, Armston J, Baker TR, Bastin JF, et al. Evaluating the potential of full-waveform lidar for mapping pan-tropical tree species richness. Glob Ecol Biogeogr. 2020;29. doi: 10.1111/geb.13158

[165] Schneider FD, Ferraz A, Hancock S, Duncanson LI, Dubayah RO, Pavlick RP, et al. Towards mapping the diversity of canopy structure from space with GEDI. Environ Res Lett. 2020;15. doi: 10.1088/1748-9326/ab9e99

[166] Hastings JH, Ollinger S V., Ouimette AP, Sanders-DeMott R, Palace MW, Ducey MJ, et al. Tree species traits determine the success of LiDAR-based crown mapping in a mixed temperate forest. Remote Sens. 2020;12. doi: 10.3390/rs12020309

[167] Singh J, Levick SR, Guderle M, Schmullius C. Moving from plot-based to hillslope-scale assessments of savanna vegetation structure with long-range terrestrial laser scanning (LR-TLS). Int J Appl Earth Obs Geoinf. 2020;90. doi: 10.1016/j.jag.2020.102070

[168] Goldbergs G, Maier SW, Levick SR, Edwards A. Efficiency of individual tree detection approaches based on light-weight and low-cost UAS imagery in Australian Savannas. Remote Sens. 2018. doi: 10.3390/rs10020161

[169] Ahmed OS, Franklin SE, Wulder MA, White JC. Characterizing stand-level forest canopy cover and height using Landsat time series, samples of airborne LiDAR, and the Random Forest algorithm. ISPRS J Photogramm Remote Sens. 2015. doi: 10.1016/j.isprsjprs.2014.11.007

[170] Hermosilla T, Ruiz LA, Kazakova AN, Coops NC, Moskal LM. Estimation of forest structure and canopy fuel parameters from small-footprint full-waveform LiDAR data. Int J Wildl Fire. 2014. doi: 10.1071/WF13086

[171] Mahlangu P, Mathieu R, Wessels K, Naidoo L, Verstraete M, Asner G, et al. Indirect estimation of structural parameters in South African forests using MISR-HR and LiDAR remote sensing data. Remote Sens. 2018. doi: 10.3390/rs10101537

[172] Windrim L, Bryson M, McLean M, Randle J, Stone C. Automated mapping of woody debris over harvested forest plantations using UAVs, high-resolution imagery, and machine learning. Remote Sens. 2019. doi: 10.3390/RS11060733

[173] Fieber KD, Davenport IJ, Tanase MA, Ferryman JM, Gurney RJ, Becerra VM, et al. Validation of Canopy Height Profile methodology for small-footprint full-waveform airborne LiDAR data in a discontinuous canopy environment. ISPRS J Photogramm Remote Sens. 2015. doi: 10.1016/j.isprsjprs.2015.03.001

[174] Gwenzi D, Lefsky MA. Modeling canopy height in a savanna ecosystem using spaceborne lidar waveforms. Remote Sens Environ. 2014. doi: 10.1016/j.rse.2013.11.024

[175] Béland M, Widlowski JL, Fournier RA, Côté JF, Verstraete MM. Estimating leaf area distribution in savanna trees from terrestrial LiDAR measurements. Agric For Meteorol. 2011. doi: 10.1016/j.agrformet.2011.05.004

[176] Tang H, Dubayah R, Swatantran A, Hofton M, Sheldon S, Clark DB, et al. Retrieval of vertical LAI profiles over tropical rain forests using waveform lidar at la selva, costa rica. Remote Sens Environ. 2012. doi: 10.1016/j.rse.2012.05.005

[177] Chen XT, Disney MI, Lewis P, Armston J, Han JT, Li JC. Sensitivity of direct canopy gap fraction retrieval from airborne waveform lidar to topography and survey characteristics. Remote Sens Environ. 2014. doi: 10.1016/j.rse.2013.12.010

[178] Xu Q, Zhang Y, Li B. Recent advances in pansharpening and key problems in applications. International Journal of Image and Data Fusion. 2014. doi: 10.1080/19479832.2014.889227

[179] Zhao P, Lu D, Wang G, Liu L, Li D, Zhu J, et al. Forest aboveground biomass estimation in Zhejiang Province using the integration of Landsat TM and ALOS PALSAR data. Int J Appl Earth Obs Geoinf. 2016;53: 1–15. doi: 10.1016/j.jag.2016.08.007

[180] Wang D, Wan B, Qiu P, Su Y, Guo Q, Wang R, et al. Evaluating the performance of Sentinel-2, Landsat 8 and Pléiades-1 in mapping mangrove extent and species. Remote Sens. 2018;10. doi: 10.3390/rs10091468

[181] Thamaga KH, Dube T. Testing two methods for mapping water hyacinth (Eichhornia crassipes) in the Greater Letaba river system, South Africa: discrimination and mapping potential of the polar-orbiting Sentinel-2 MSI and Landsat 8 OLI sensors. Int J Remote Sens. 2018;39: 8041–8059. doi: 10.1080/01431161.2018.1479796

[182] Pohl C, van Genderen J. Remote sensing image fusion: An update in the context of Digital Earth. International Journal of Digital Earth. 2014. doi: 10.1080/17538947.2013.869266

[183] Piovan SE. Remote Sensing. Springer Geography. 2020. doi: 10.1007/978-3-030-42439-8_7

[184] Attarchi S, Gloaguen R. A multi-sensor approach for improving biodiversity estimation in the Hyrcanian mountain forest, Iran. Int J Remote Sens. 2018;39: 7311–7327. doi: 10.1080/01431161.2018.1468114

[185] Alparone L, Aiazzi B, Baronti S, Garzelli A. Remote Sensing Image Fusion. Remote Sensing Image Fusion. 2015. doi: 10.1201/b18189

[186] Lu M, Chen B, Liao X, Yue T, Yue H, Ren S, et al. Forest types classification based on multi-source data fusion. Remote Sens. 2017;9. doi: 10.3390/rs9111153

[187] Plank S, Jüssi M, Martinis S, Twele A. Mapping of flooded vegetation by means of polarimetric sentinel-1 and ALOS-2/PALSAR-2 imagery. Int J Remote Sens. 2017;38. doi: 10.1080/01431161.2017.1306143

[188] Kattenborn T, Maack J, Faßnacht F, Enßle F, Ermert J, Koch B. Mapping forest biomass from space—Fusion of hyperspectralEO1-hyperion data and Tandem-X and WorldView-2 canopy heightmodels. Int J Appl Earth Obs Geoinf. 2015. doi: 10.1016/j.jag.2014.10.008

[189] Ali MZ, Qazi W, Aslam N. A comparative study of ALOS-2 PALSAR and landsat-8 imagery for land cover classification using maximum likelihood classifier. Egypt J Remote Sens Sp Sci. 2018;21: S29–S35. doi: 10.1016/j.ejrs.2018.03.003

[190] Rajah P, Odindi J, Mutanga O. Feature level image fusion of optical imagery and Synthetic Aperture Radar (SAR) for invasive alien plant species detection and mapping. Remote Sens Appl Soc Environ. 2018;10: 198–208. doi: 10.1016/j.rsase.2018.04.007

[191] Held A, Ticehurst C, Lymburner L, Williams N. High resolution mapping of tropical mangrove ecosystems using hyperspectral and radar remote sensing. Int J Remote Sens. 2003;24. doi: 10.1080/0143116031000066323

[192] Hartling S, Sagan V, Sidike P, Maimaitijiang M, Carron J. Urban tree species classification using a worldview-2/3 and liDAR data fusion approach and deep learning. Sensors (Switzerland). 2019;19. doi: 10.3390/s19061284 30875732 PMC6471063

[193] Colgan MS, Baldeck CA, Féret J baptiste, Asner GP. Mapping savanna tree species at ecosystem scales using support vector machine classification and BRDF correction on airborne hyperspectral and LiDAR data. Remote Sens. 2012. doi: 10.3390/rs4113462

[194] Mayr MJ, Samimi C. Comparing the dry season in-situ leaf area index (lai) derived from high-resolution rapideye imagery with MODIS LAI in a namibian savanna. Remote Sens. 2015;7. doi: 10.3390/rs70404834

[195] Pastick NJ, Wylie BK, Wu Z. Spatiotemporal analysis of Landsat-8 and Sentinel-2 data to support monitoring of dryland ecosystems. Remote Sens. 2018;10. doi: 10.3390/rs10050791

[196] Adhikari H, Valbuena R, Pellikka PKE, Heiskanen J. Mapping forest structural heterogeneity of tropical montane forest remnants from airborne laser scanning and Landsat time series. Ecol Indic. 2020. doi: 10.1016/j.ecolind.2019.105739

[197] Swatantran A, Dubayah R, Roberts D, Hofton M, Blair JB. Mapping biomass and stress in the Sierra Nevada using lidar and hyperspectral data fusion. Remote Sens Environ. 2011;115. doi: 10.1016/j.rse.2010.08.027

[198] Qi W, Dubayah RO. Combining Tandem-X InSAR and simulated GEDI lidar observations for forest structure mapping. Remote Sens Environ. 2016;187. doi: 10.1016/j.rse.2016.10.018

[199] Lee SK, Fatoyinbo T, Marselis SM, Qi W, Hancock S, Armston J, et al. Spaceborne Data Fusion for Large-Scale Forest Parameter Estimation: GEDI Lidar and Tandem-X INSAR Missions. International Geoscience and Remote Sensing Symposium (IGARSS). 2019. doi: 10.1109/IGARSS.2019.8899854

[200] Pourshamsi M, Garcia M, Lavalle M, Balzter H. A Machine-Learning Approach to PolInSAR and LiDAR Data Fusion for Improved Tropical Forest Canopy Height Estimation Using NASA AfriSAR Campaign Data. IEEE J Sel Top Appl Earth Obs Remote Sens. 2018;11. doi: 10.1109/JSTARS.2018.2868119

[201] Khare S, Latifi H, Ghosh SK. Multi-scale assessment of invasive plant species diversity using Pléiades 1A, RapidEye and Landsat-8 data. Geocarto Int. 2018;33: 681–698. doi: 10.1080/10106049.2017.1289562

[202] Urban M, Heckel K, Berger C, Schratz P, Smit IPJ, Strydom T, et al. Woody cover mapping in the savanna ecosystem of the Kruger National Park using sentinel-1 C-band time series data. Koedoe. 2020. doi: 10.4102/koedoe.v62i1.1621

[203] Sun Y, Huang J, Ao Z, Lao D, Xin Q. Deep learning approaches for the mapping of tree species diversity in a tropical wetland using airborne LiDAR and high-spatial-resolution remote sensing images. Forests. 2019. doi: 10.3390/F10111047

[204] Forkuor G, Conrad C, Thiel M, Landmann T, Barry B. Evaluating the sequential masking classification approach for improving crop discrimination in the Sudanian Savanna of West Africa. Comput Electron Agric. 2015. doi: 10.1016/j.compag.2015.09.020

[205] Mathieu R, Naidoo L, Cho MA, Leblon B, Main R, Wessels K, et al. Toward structural assessment of semi-arid African savannahs and woodlands: The potential of multitemporal polarimetric RADARSAT-2 fine beam images. Remote Sens Environ. 2013. doi: 10.1016/j.rse.2013.07.011

[206] Mitchell JJ, Shrestha R, Spaete LP, Glenn NF. Combining airborne hyperspectral and LiDAR data across local sites for upscaling shrubland structural information: Lessons for HyspIRI. Remote Sens Environ. 2015. doi: 10.1016/j.rse.2015.04.015

[207] Kattenborn T, Lopatin J, Förster M, Braun AC, Fassnacht FE. UAV data as alternative to field sampling to map woody invasive species based on combined Sentinel-1 and Sentinel-2 data. Remote Sens Environ. 2019. doi: 10.1016/j.rse.2019.03.025

