# Supplementary material for: Remote sensing of savanna woody species diversity: A systematic review of data types and assessment methods
Source: PLoS One. 2022 Dec 1;17(12):e0278529. doi: 10.1371/journal.pone.0278529 (PMC9714920; doi:10.1371/journal.pone.0278529)
Supplement: S1 Table — (PDF) [file pone.0278529.s002.pdf]

AMSTAR 2 - Question content

1. Did the research questions and inclusion criteria for the review include the components of PICO?
2. Did the report of the review contain an explicit statement that the review methods were established prior to the conduct of the review and did the report justify any significant deviations from the protocol?
3. Did the review authors explain their selection of the study designs for inclusion in the review?
4. Did the review authors use a comprehensive literature search strategy?
5. Did the review authors perform a study selection?
6. Did the review authors perform data extraction in duplicate?
7. Did the review authors provide a list of excluded studies and justify the exclusions?
8. Did the review authors describe the included studies in adequate detail?
9. Did the review authors use a satisfactory technique for assessing the risk of bias (RoB) in individual studies that were included in the review?
10. Did the review authors report on the sources of funding for the studies included in the review?
11. If meta-analysis was performed, did the review authors use appropriate methods for statistical combination of results?
12. If meta-analysis was performed, did the review authors assess the potential impact of RoB in individual studies on the results of the meta-analysis or other evidence synthesis?
13. Did the review authors account for RoB in primary studies when interpreting/discussing the results of the review?
14. Did the review authors provide a satisfactory explanation for, and discussion of, any heterogeneity observed in the results of the review?
15. If they performed quantitative synthesis did the review authors carry out an adequate investigation of publication bias (small study bias)and discuss its likely impact on the results of the review?
16. Did the review authors report any potential sources of conflict of interest, including any funding for conducting the review?

Evaluation (Yes: 1 point; PY: 0.5 point; No: 0 point). Aggregated Score Analysis: Critically low quality, low quality, moderate quality, high quality important items 2, 4, 7, 9, 11. If one item is omitted, it is rated as excellent quality. In the case of AMSTAR 2, depending on the number of important items missing out of all16 questions (items 2, 4, 7, 9, 11, 13, 15), the assessment is as follows: “Critically low quality,” “Low quality,” “Moderate quality,” and “High quality”. AMSTAR 2 has the advantage of being able to establish specific and clear results for instance it does not use“Partial Yes”, rather however there is use of “Yes” and “No.”

Evaluation (Yes: 1 point, No: 0 point, Not applicable: 0 point). Aggregated Score Analysis (0–4 points: Low level;5–8 points: Moderate level; 9–11 points: High level).

Supplemental Table. Quality assessment scores based on AMSTAR2 for review of reviews																
**First author- Year - Review Topic**	**Criteria**															
	**1**	**2**	**3**	**4**	**5**	**6**	**7**	**8**	**9**	**10**	**11**	**12**	**13**	**14**	**15**	**16**
Karlson 2016 Remote sensing of vegetation in the Sudano-Sahelian zone	N	N	Y	Y	Y	Y	N	N	N	Y	N	N	N	Y	Y	Y
Moore 2016 Australian vegetation phenology	N	N	Y	Y	Y	Y	N	N	N	Y	N	N	N	Y	Y	Y
Pohl 1998 Multisensor image fusion in remote sensing	N	N	Y	Y	Y	Y	N	N	N	Y	N	N	N	Y	Y	Y
Maxwell 2018 Implementation of machine-learning classification	N	N	Y	Y	Y	Y	N	N	N	Y	N	N	N	Y	Y	Y
Adole 2016 Vegetation phenology in Africa	N	N	Y	Y	Y	Y	N	N	N	Y	N	N	N	Y	Y	Y
Ganivet 2019 Assessments of tree species diversity and structure	N	N	Y	Y	Y	Y	N	N	N	Y	N	N	N	Y	Y	Y
Gardon 2020 Brazil's forest restoration, biomass and carbon stocks	N	N	Y	Y	Y	Y	N	N	N	Y	N	N	N	Y	Y	Y
Mutanga 2016 Vegetation monitoring in South Africa	N	N	Y	Y	Y	Y	N	N	N	Y	N	N	N	Y	Y	Y
Santos 2016 Remote sensing in the study of Brazilian vegetation	N	N	Y	Y	Y	Y	N	N	N	Y	N	N	N	Y	Y	Y
De Menezes 2008 Studies in the Amazonian environment	N	N	Y	Y	Y	Y	N	N	N	Y	N	N	N	Y	Y	Y
Fassnacht 2016 Tree species classification from remotely sensed data	N	N	Y	Y	Y	Y	N	N	N	Y	N	N	N	Y	Y	Y
Xie 2008 Remote sensing imagery in vegetation mapping	N	N	Y	Y	Y	Y	N	N	N	Y	N	N	N	Y	Y	Y
Shen 2019 Mapping by synthetic aperture RADAR	N	N	Y	Y	Y	Y	N	N	N	Y	N	N	N	Y	Y	Y
Zhu 2018 Remote Sensing Sensors	N	N	Y	Y	Y	Y	N	N	N	Y	N	N	N	Y	Y	Y
Kulkarni 2020 Fusion, SAR and optical images	N	N	Y	Y	Y	Y	N	N	N	Y	N	N	N	Y	Y	Y
Hyyppä 2008 Methods of small-footprint airborne laser scanning for extracting forest	N	N	Y	Y	Y	Y	N	N	N	Y	N	N	N	Y	Y	Y
Wulder 2012 Lidar sampling for large-area forest characterization	N	N	Y	Y	Y	Y	N	N	N	Y	N	N	N	Y	Y	Y
